# The heterogeneity of mammalian utricular cells over the course of development

**DOI:** 10.1002/ctm2.1052

**Published:** 2022-09-30

**Authors:** Dan You, Jin Guo, Yunzhong Zhang, Luo Guo, Xiaoling Lu, Xinsheng Huang, Shan Sun, Huawei Li

**Affiliations:** ^1^ ENT Institute and Otorhinolaryngology Department of Eye & ENT Hospital, State Key Laboratory of Medical Neurobiology and MOE Frontiers Center for Brain Science Fudan University Shanghai China; ^2^ Department of Otorhinolaryngology‐Head and Neck Surgery Zhongshan Hospital Fudan University Shanghai China; ^3^ Institutes of Biomedical Sciences Fudan University Shanghai China; ^4^ NHC Key Laboratory of Hearing Medicine, Fudan University Shanghai China; ^5^ The Institutes of Brain Science and the Collaborative Innovation Center for Brain Science Fudan University Shanghai China

**Keywords:** epithelial non‐hair cell, hair cell differentiation, inner ear development, mitotic cell proliferation, single‐cell RNA sequencing, Sox9‐CreER mouse, supporting cell, utricle

## Abstract

**Background:**

The inner ear organ is a delicate tissue consisting of hair cells (HCs) and supporting cells (SCs).The mammalian inner ear HCs are terminally differentiated cells that cannot spontaneously regenerate in adults. Epithelial non‐hair cells (ENHCs) in the utricle include HC progenitors and SCs, and the progenitors share similar characteristics with SCs in the neonatal inner ear.

**Methods:**

We applied single‐cell sequencing to whole mouse utricles from the neonatal period to adulthood, including samples from postnatal day (P)2, P7 and P30 mice. Furthermore, using transgenic mice and immunostaining, we traced the source of new HC generation.

**Results:**

We identified several sensory epithelial cell clusters and further found that new HCs arose mainly through differentiation from Sox9+ progenitor cells and that only a few cells were produced by mitotic proliferation in both neonatal and adult mouse utricles. In addition, we identified the proliferative cells using the marker UbcH10 and demonstrated that in adulthood the mitotically generated HCs were primarily found in the extrastriola. Moreover, we observed that not only Type II, but also Type I HCs could be regenerated by either mitotic cell proliferation or progenitor cell differentiation.

**Conclusions:**

Overall, our findings expand our understanding of ENHC cell fate and the characteristics of the vestibular organs in mammals over the course of development.

## Graphical abstract

Single‐cell RNA‐seq identified several cell clusters in whole mouse utricle sensory epithelia from the neonatal period to adulthood. New HCs mainly differentiated from Sox9 progenitor cells, and only a few were produced by mitotic proliferation. Ubch10 cells could generate HCs mitotically in adulthood.

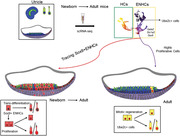

## 1 INTRODUCTION

The mammalian utricle, which has two anatomical zones, namely the central striola and the surrounding extrastriola,^1^, ^2^ is an otolithic organ that responds to horizontal acceleration and head tilt with respect to gravity.^3^ The mature utricular sensory epithelium consists of two primary cell types, namely hair cells (HCs) and supporting cells (SCs), both of which appear to be grossly homogeneous. HCs in the sensory epithelial areas of the vestibule can be divided into Type I and Type II HCs. Type I HCs have a flask‐like body that forms synapses with large calyx afferent fibres. Type II HCs have thick necks and basal cytoplasmic processes projecting from the cell body, and they synapse onto bouton afferent nerves.^4^, ^5^, ^6^ Moreover Type I HCs located in the centre of the sensory epithelium express a low‐voltage‐activated outward K^+^ current, *I*_K,L_.^7^ K+ accumulates in the synaptic cleft around Type I HCs and depolarizes both the pre‐ and postsynaptic membranes making Type I HCs more suitable for detecting the acceleration of head movement.^8^ The transcription factor Sox2 and calcium binding protein are differentially expressed in the two types of HCs^9^, ^10^, ^11^ and thus are often used as reliable markers to distinguish the two types of HCs. However, there is evidence that SCs might be involved in heterogeneous functions, including HC regeneration and/or differentiation.^12^, ^13^

Utricular HCs are the mechanoreceptors of balance, and about half of these cells in adult mice arise during the first 2 weeks after birth. It has been shown that utricular HCs arise more sporadically over an extended period that spans from embryonic day (E)13 to postnatal day (P)12.^3^, ^13^, ^14^, ^15^ The expanded utricular HCs can originate either as new cells arising from the division of progenitor cells,^16^, ^17^, ^18^, ^19^ which is also called mitotic differentiation,^20^ or by direct differentiation in which progenitor cells convert into HCs in early developmental stages without first undergoing mitotic division.^12^, ^21^, ^22^, ^23^ Furthermore, unlike cochlear cells, mammalian utricular HCs have limited turnover or regeneration capacity after damage in adulthood.^7^, ^24^, ^25^ The newly generated HCs can be captured with a transitional appearance and immature morphology,^7^, ^26^, ^27^ which indicates that progenitor cells should be present during the postnatal period. However, the sources of both types of regenerated HCs have been poorly explored.

The utricular progenitor cells, which share similar morphological and histochemical characteristics with SCs, can proliferate neonatally, but this capacity is rapidly lost after birth.^28^ The distinctions between these progenitors and other SCs are difficult to determine using bulk RNA‐seq or histochemical immunostaining. In a previous study, Burns et al.^13^ isolated HCs and SCs from Gfi1+ and Lfng+ fluorescent transgenic mice and further analysed their transcription by single‐cell RNA sequencing (scRNA‐seq). They identified transitional epithelial cells, SCs, HCs and transitional cells between SCs and HCs in the utricles, which further indicated the heterogeneity of the utricular cells. Another study by Elkon et al.^29^ defined vestibular epithelia cells as HCs, epithelial non‐HCs (ENHCs) and non‐epithelial cells. The ENHCs include transitional epithelia cells, SCs and other non‐HCs in the utricular epithelium. However, the roles of individual ENHC subpopulations have not been determined.

In the present study, we investigated mouse utricle cells using scRNA‐seq, and we identified ENHCs by marker genes and then characterized them into several sub‐populations. Based on the lineage tracing of transgenic mouse strains, we found that most of the newly generated HCs originated from the direct differentiation of Sox9+ cells. In addition, only a small portion of HCs were newly produced from UbcH10+ cells, which corresponded to the previously reported novel cell population in the mouse cochlea.^30^ Our results show the heterogeneity of utricular ENHCs and provide direct evidence for the regenerative capacity of vestibular ENHCs and suggest a mechanism through which adult mammalian HCs might regenerate in the inner ear.

## 2 RESULTS

### 2.1 scRNA‐seq of neonatal mouse utricles and the heterogeneity of progenitor cells

**FIGURE 1:**
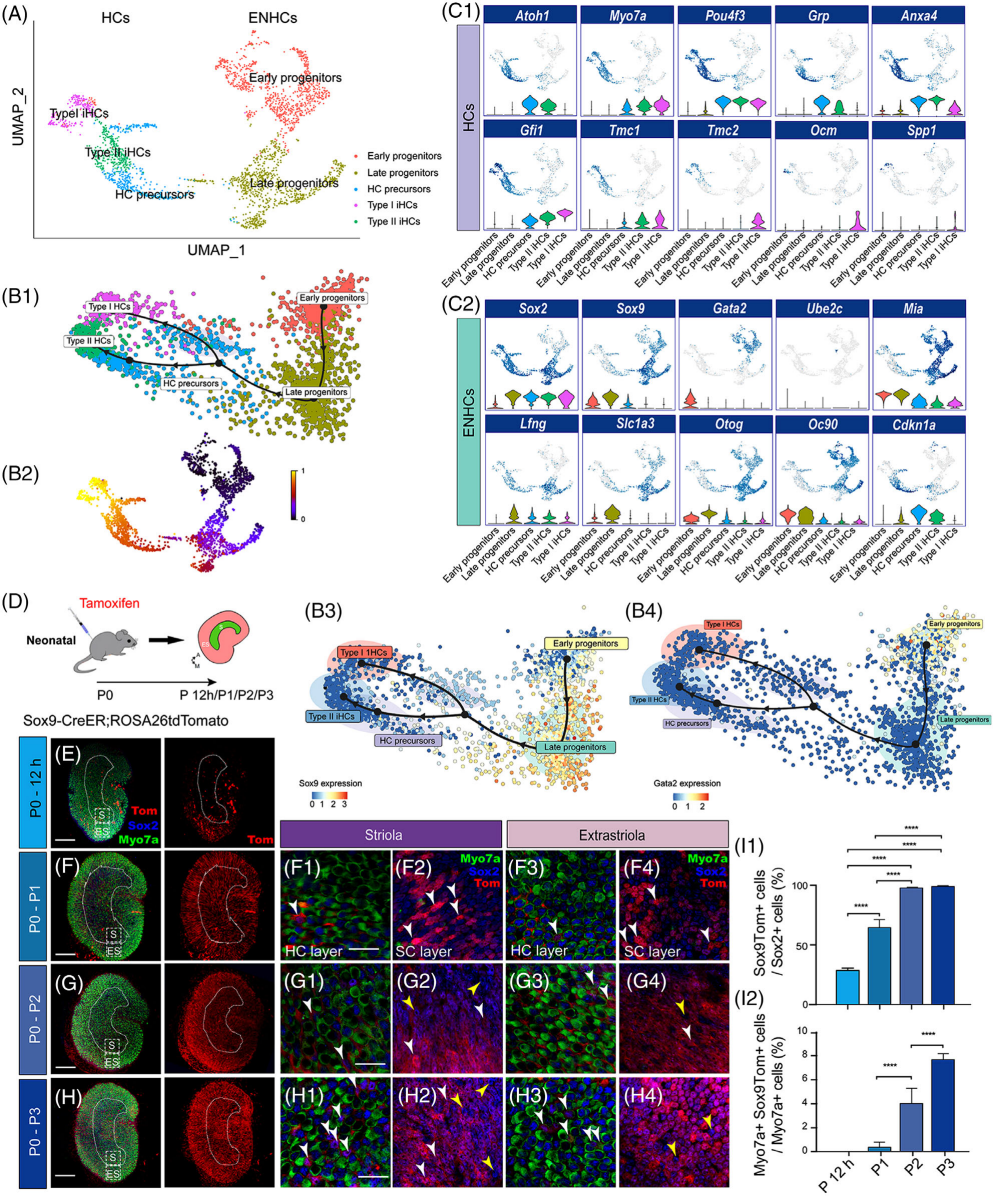
The subtypes of mouse utricular sensory epithelial cells in the neonatal period. (A) UMAP clustering of HCs, progenitors and precursors from P2 mouse utricle sensory epithelium. Single cells are coloured by cell types, including Early progenitors (early‐stage HC progenitors), Late progenitors (late‐stage HC progenitors), HC precursors, Type I HCs, and Type II HCs. (B1–B4) Cell trajectory analysis of the P2 cell subset (B1) Slingshot trajectory showing the ordering of single cells in pseudotime. Cell clusters are indicated by different colours. (B2) Dynamical model reconstruction through RNA velocity analysis and latent time recovery of potent cellular processes. Underlying time processes are indicated by colour changes. (B3,B4) *Sox9* and *Gata2* mRNA expression levels in the Slingshot trajectory. (C1,C2) Visualization of gene expression in UMAP dimension‐reduced space using feature plots and violin plots. (C1) The expression levels of P2 mouse utricular HC markers. (C2) Visualization of the expression levels of other ENHC markers in the P2 mouse utricle sensory epithelium. (D) Diagram describing the procedure of tamoxifen administration in Sox9tdTomato mice. (E) At 12 h after tamoxifen injection, only 28.62% ± 0.86% of the Sox2+ progenitor cells expressed red tdTomato fluorescent protein in P0 mice. (F; F1–F4) A total of 64.62% ± 2.67% of the Sox2+ progenitor cells expressed red fluorescent protein at 24 h after tamoxifen injection. (G, G1–G4; H, H1–H4) A total of 97.64% ± 0.28% (48 h after tamoxifen injection) and 99.09% ± 0.23% (72 h after tamoxifen injection) Sox2+ progenitor cells expressed red fluorescent protein. The white arrow in e1 through g4 indicates tdTomato+ cells. The yellow arrow in e1 through g4 indicates tdTomato− cells. (I1,I2) Quantification and comparison of the recombination efficiency of the Sox9+ progenitors (I1) and the proportion of Myo7a+/tdTomato+ HCs at different time points after tamoxifen application (I2). Scale bars are 100 µm in D, E, F, and G and 20 µm in E1, F1 and G1. Data are shown as the mean ± SD; student's *t*‐test, ** *p* < .01, **** *p* < .0001, *n* = 3

Previous studies focused on special cell populations marked by fluorescent proteins, by which inner ear sensory epithelial cells were marked and selected by flow cytometry.^13^ Here, we carried out scRNA‐seq experiments with cells obtained from whole utricular epithelia (Figure S1A,B) from both sexes of P2 C57/B6 mice in order to molecularly profile the utricular epithelial cells. In the Uniform Manifold Approximation and Projection (UMAP) dimensionality reduction plot, we divided all cells into immature HCs and ENHCs based on the expression of marker genes and pseudotime analysis (Figure 1A,B1–4). The cell quality was satisfactory for our scRNA‐seq experiments (Figure S1C). The immature HCs expressed relatively high levels of *Myo7a*, *Anxa4*, *Atoh1*, *Pou4f3* and *Gfi1*(Figure 1C1), some of which, such as *Atoh1* and *Pou4f3*, have been reported in neonatal mice.^31^, ^32^, ^33^ The inner ear‐specific glycoprotein Otog, which continuously processes otogelin,^34^ and the predominant mammalian otoconial protein Oc90^35^ overlapped with ENHCs (Figure 1C2 and Figure S1E). Sox2 was highly expressed in late progenitors, HC precursors and immature HCs, but was only expressed at low levels in early progenitors. However, ENHCs expressed unique markers, including *Gata2*, *Slc1a3*(protein name: GLAST, Figure S1D) and *Sox9* (Figure 1C1,C2). As a “vestibular gene,” *Gata2* has been reported to be involved in the fine tuning of ear morphogenesis.^36^, ^37^ Cluster analysis revealed highly differentially expressed genes, and Slingshot trajectory analysis was used for identifying progenitor cell differentiation, the results of which indicated that *Gata2*+ cells might include but not be limited to early progenitors during the neonatal stage (Figure 1B1–B4). Besides *Gata2*, other genes, including *Pla2g7*, *Cnmd*, *Gpc3* and so forth, were also found in the same cluster of cells (Figure S1F). Of the GATA factors, sustained expression of *Gata3* is required to fully differentiate HCs and spiral ganglion neurons in the organ of Corti.^38^

### 2.2 Lineage tracing of utricular progenitor cells

Sox9 is a useful SC marker in the utricles^39^ and is highly expressed in HC precursors and progenitors. Here, Sox9‐CreERT2; ROSA26‐tdTomato mice were used to trace the fate of Sox9+ progenitors and to explore their roles in sensory cell development (Figure 1D). In the neonatal P2 mice, we found that the ratio of Sox9tdTomato+ cells to the total Sox2+ cells increased with time. Meanwhile, a small number of Myo7a+/Sox9tdTomato+ cells were detected in the HC layer, and the number increased with time following tamoxifen administration (Figure 1E–H,I1,I2), while no Myo7a+/Sox9tdTomato+ cells were detected in the Sox9tdTomato transgenic mice treated with saline (Figure S2A,B). The expression of Sox9 in the non‐sensory epithelium was also analysed and compared with the expression of Sox2 in P0–P1 mice (Figure S2C–E). These results indicated that during the early development stage Sox9‐CreERT2‐mediated recombination resulted in the vast majority of the ENHCs expressing tdTomato within 48 h after injection of tamoxifen, and some of them differentiated into HCs soon after.

### 2.3 ENHC tracing in developing utricles

**FIGURE 2:**
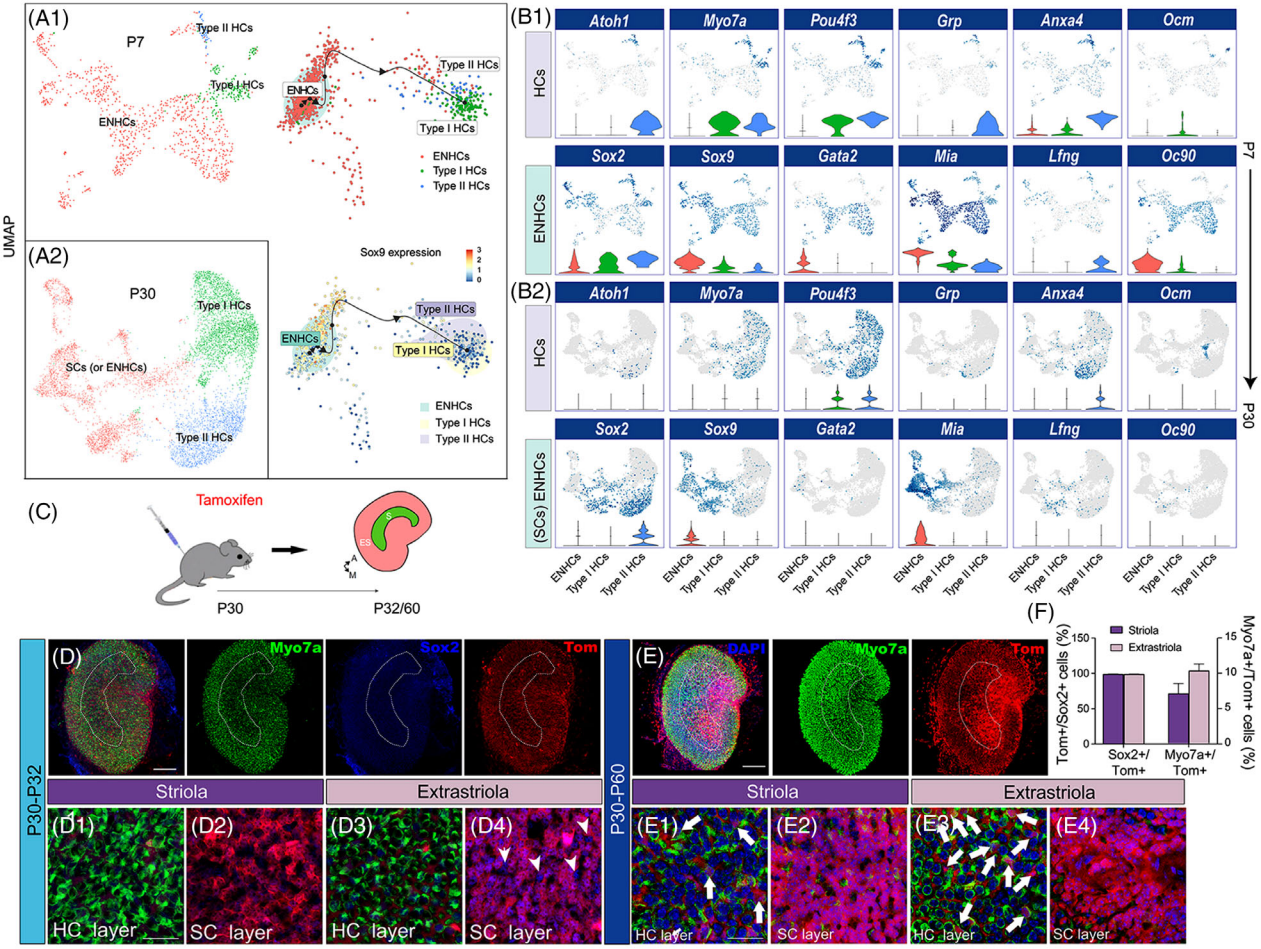
The cell population of mouse utricle sensory epithelial cells at the postnatal development stage and mature stage. (A1) UMAP clustering of HCs and ENHCs in P7 mouse utricle sensory epithelium. The inserted plots show the trajectory analysis results of P7 HCs and ENHCs using Slingshot. Expression levels of Sox9 were quantified across the pseudotime. (A2) UMAP mapping of HCs and SCs in P30 mouse utricle sensory epithelium. (B1,B2) Gene features of HCs and ENHCs from P7 and P30. (C) A schematic diagram depicting the tracing procedure. S: striola; ES: extrastriola. The two arrows indicate the anatomical orientations (A = anterior, M = medial). (D; D1–4) Two days after the injection of tamoxifen into P30 Sox9‐CreER; tdTomato mice, most of the ENHCs expressed tdTomato fluorescent protein except for the cells indicated by arrowheads (D4). Arrowheads in E4 indicate tdTomato− cells. (E; E1–4) One month after tamoxifen injection, almost all of the ENHCs in the extrastriola expressed tdTomato fluorescent protein (E4). White arrows in E1 and E2 indicate Myo7a+/tdTomato+ double‐positive cells. (F) Quantification of tdTomato+ cells and the Myo7a+/tdTomato+ cell proportion in Sox9‐CreER;tdTomato mice. Tom, tdTomato. Scale bars are 100 µm in D and E and 20 µm in D1 and E1

In order to better understand the characteristics of developing vestibular ENHCs, we further dissected utricles from P7 and P30 mice (Figure 2A1,2). In the Slingshot analysis, only one trajectory was identified from ENHCs to HCs at P7, and there were no trajectory paths identified in P30 mice. Furthermore, gene expression levels indicated that several marker genes, including *Gata2*, *Mia* and *Sox9*, undergo large changes during development (Figure 2B1,2). Cell type‐specific markers across all clusters called in the UMAP plot are listed in Figure S3. It was interesting that at P7 *Sox9* could be detected in most subtypes of sensory epithelial cells; however, at P30, Sox9 was again mainly expressed in ENHCs, similar to its expression pattern at P2(Figure 2B1,2). These results for the Sox9 expression pattern during development were validated with immunostaining (Figure S2F–H).

By lineage tracing in adult P30 mice (Figure 2C), we tried to define the cell fate of Sox9+ ENHCs in adult mice. At 48 h after tamoxifen injection, Sox9tdTomato was present in most of the Sox2+ cells in the transgenic mice (Figure 2D–D4), especially in the striola, while there were some Sox2+/Sox9− cells in the extrastriola. When utricles were harvested one month after tamoxifen administration, most tdTomato+ cells in the HC layer were Myo7a+ cells (Figure 2E–E4,F). Even in P60 mice the Cre recombinase could be fully activated in Sox9 transgenic mice within 72 h following tamoxifen administration (Figure S2I). In addition, relatively lower levels of newly generated HCs from Sox9tdTomato‐sourced cells were observed in the adult utricles compared to those in the neonatal utricles (Figure 2E1–E4,F). These results indicated that even in adults Sox9+ ENHCs could still be potential source for HC generation. However, the turnover rate was only about 8%‐10%, and this was maintained at a low level.

### 2.4 Heterogeneity of ENHCs in adult utricles

**FIGURE 3:**
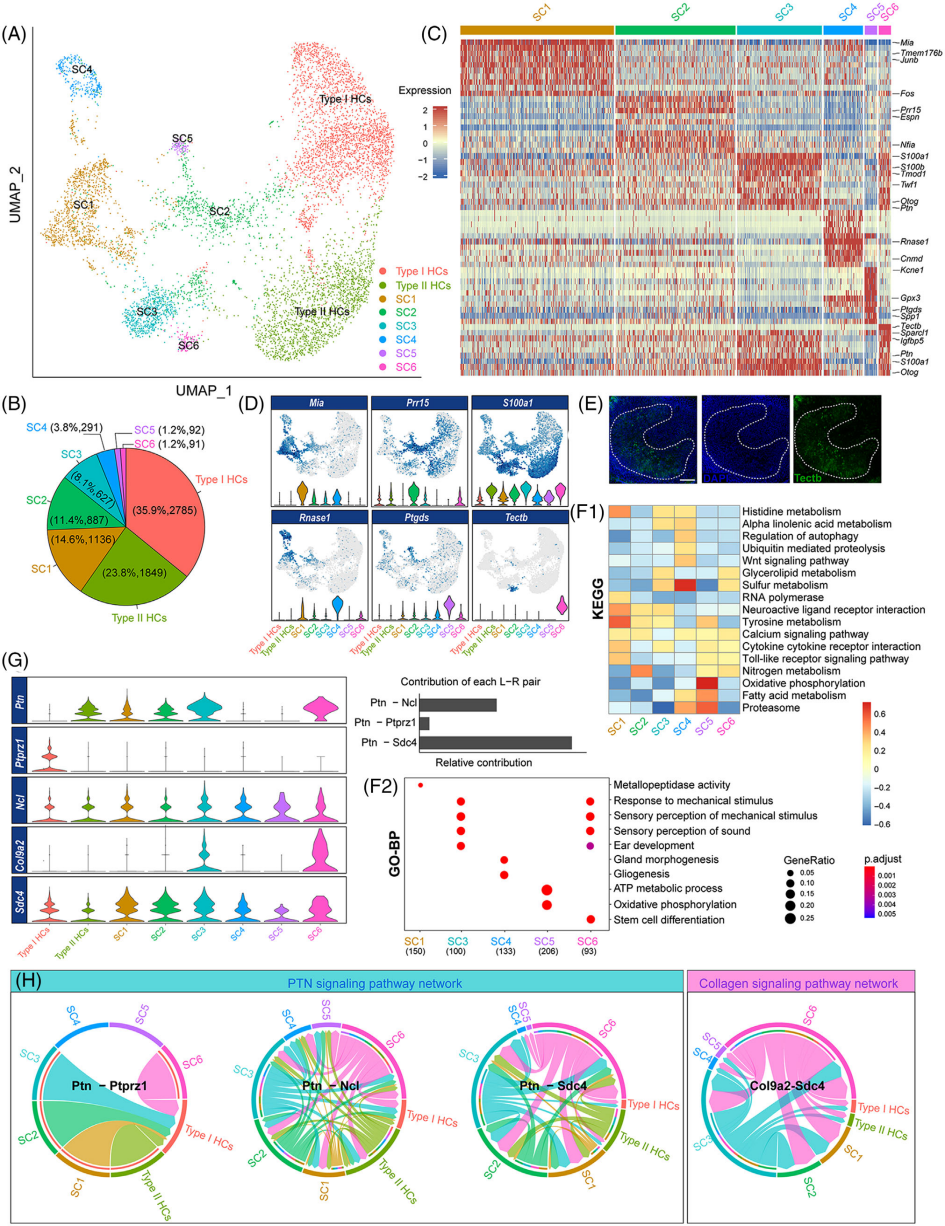
The heterogeneity of HCs and SCs from P30 mouse utricle sensory epithelium. (A) UMAP clustering of HCs and SCs in the P30 mouse utricle sensory epithelium. Type I HCs, Type II HCs, and six different SC populations are labelled with different colours. (B) The proportions of different cell clusters in P30 utricle sensory epithelium illustrated by a pie chart. (C) Heatmap showing the differentially expressed genes in the P30 SC clusters. (D) Feature plots and violin plots showing the informative genes in each cluster. (E) Immunofluorescent staining of DAPI and Tectb in P30 mouse utricles. The striolar boundaries are labelled as dotted lines. (F1) The comparison of KEGG pathway annotation between different SC clusters. The top categories are shown. (F2) Gene ontology (GO) analyses of SC clusters at P30. GO subitems specifying the biological process (GO‐BP) are displayed. (G,H) Inference of potential cell–cell communication networks for P30 HC and SC datasets using CellChat. (G) Violin plot of signalling gene expression related to the most active signalling pathways according to CellChat. The inserted bar plot on the right shows the contribution of each ligand‐receptor pair to the overall effect of the PTN signalling pathway. (H) Chord diagrams divided by individual ligand‐receptor pairs were used for visualizing the cell–cell communication networks. Arrows point to potential signal recipient cells

In order to further characterize the cell types in the P30 mammalian utricles in addition to the two groups of HCs, the 3124 cells in the ENHC group were divided into six groups of SCs that were notably characterized by the expression of specific genes (Figure 3A–C), including *Mia*, *Prr15*, *Ptgds*, *Rnase1*, *Tectb*, and so forth (Figure 3D). Of note, some marker genes were exclusively expressed in specific clusters. For example, *Tectb*, which codes for the otolith membrane glycoprotein β‐tectorin, was specifically expressed in SC6 in the striola (Figure 3E).^13^ We next analysed cell functions by KEGG/GO analysis and CellChat, and the subtypes of SCs showcased different functions (Figure 3F1,2).

CellChat detected two significant ligand‐receptor pairs among the eight cell groups (including two groups of HCs and six groups of ENHCs), namely the PTN signalling pathway and the collagen signalling pathway (Figure 3G,H). However, in contrast with P30 mice there were dozens of signalling pathways identified in neonatal (P2) mice, including the CDH, Notch and non‐canonical WNT signalling pathways (Figure S4). The functions of individual clusters were different as shown in Figure S5. In the neonatal stage, Notch signal transduction mainly occurred between HCs and progenitors, which was in accordance with previous observations.^18^

### 2.5 Cell fate tracing of utricular ENHCs

**FIGURE 4:**
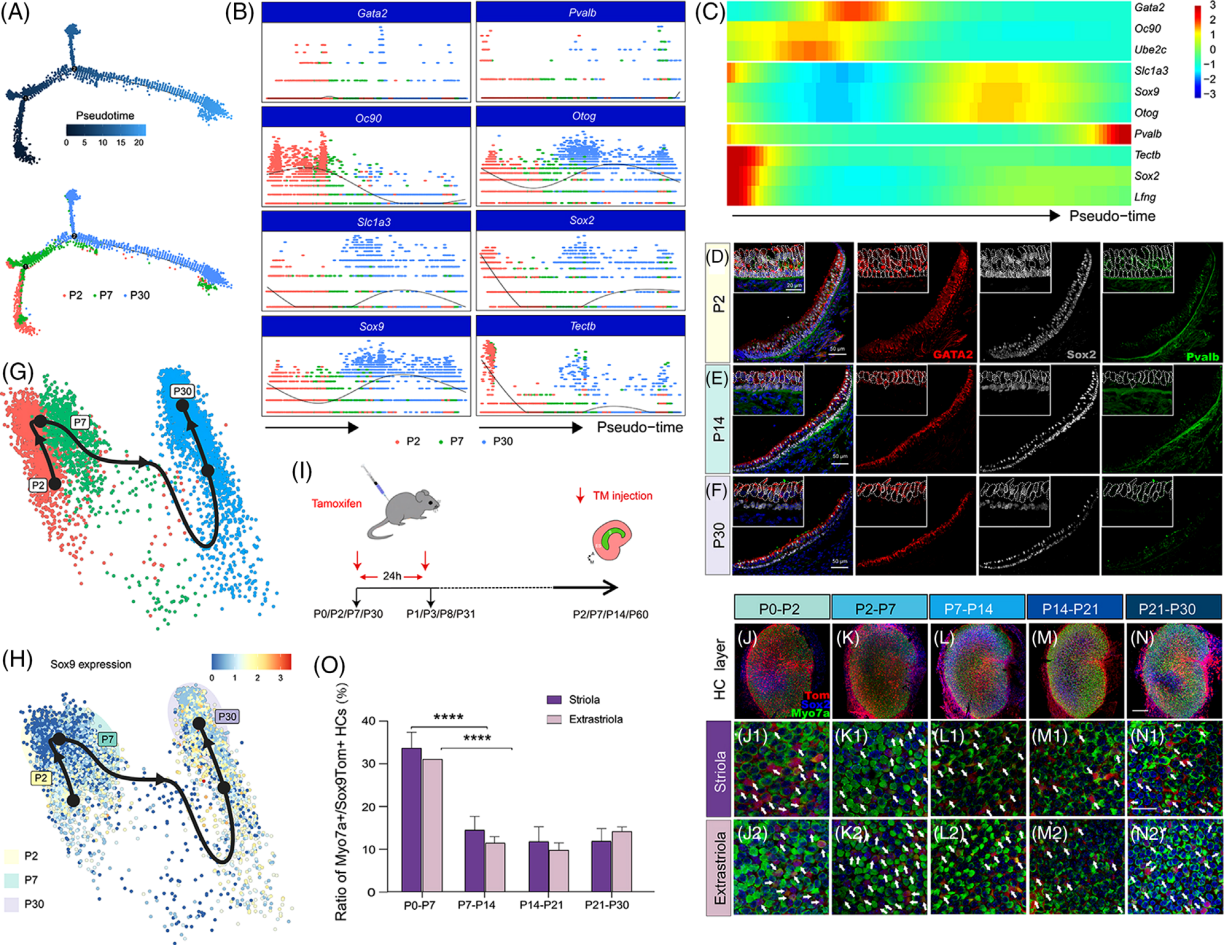
Lineage tracing of Sox9+ cells at different time points. (A–D) Single‐cell analysis of a combined dataset. Progenitor cells at P2, ENHCs at P7, and SCs at P30, which were annotated previously, were integrated. Batch effects between single‐cell datasets at different time points were eliminated using the Harmony integration function. (A) Monocle was used to generate pseudotime trajectories in the combined cell datasets at different time points. Cells were labelled along the trajectory by pseudotime (upper) and according to actual development time point (lower). (B) Plotting the expression levels of highly variable gene subsets along the pseudotime axis. The cell attribute is displayed as the original time point with different colours. (C) Heatmap used to trace the differentially expressed genes along the pseudotime axis. (D–F) Laser confocal scanning of utricle transverse sections at different time points. Cells were stained with antibodies against Gata2 (red), Sox2 (grey) and parvalbumin (green). (G,H) Slingshot trajectory analysis of combined cell datasets at different time points. Single cells were labelled by primitive time points (G) and Sox9 mRNA expression levels (H). (I) Diagram describing the procedure of tamoxifen administration in Sox9+/tdTomato+ mice. (J–N) Newborn Myo7a+/tdTomato+ HCs (labelled with arrows) were detected in the striola and extrastriola at different time periods, including P0–P2 (J–J2), P2–P7 (K–K2), P7–P14 (L–L2), P14–P21 (M–M2), and P21–P30 (N–N2). (O) Histogram illustrating the percentage of regenerated HCs at different time periods in the striola and extrastriola. Data are shown as the mean ± SD; student's *t*‐test, ****p < .0001, *n* = 3. Scale bars are 100 µm in N, 50 µm in D, E, F; 20 µm in N1; and 10 µm in the top left corner enlarged view in D

We next pooled all of the ENHCs from P2, P7 and P30 utricular sensory epithelium using Harmony and performed a pseudotime gradient using Monocle (Figure 4A). This revealed the expression profiles of several genes, including downregulated genes (*Gata2*, *Oc90*) and some dynamically expressed genes, such as *Sox2*, *Sox9* and *Tectb* (Figure 4B,C). The *Gata2* gene decreased over time as confirmed by immunostaining (Figure 4D–F). Slingshot trajectory analysis of combined cell datasets from different timepoints again indicated that *Sox9* was dynamically expressed and was further concentrated in ENHCs in adult mice (Figure 4G,H).

We used Sox9tdTomato transgenic mice to quantify the capacity of Sox9+ cells to differentiate into HCs in the utricles during postnatal development. The mice were divided into three stages—neonatal (P0–P2), juvenile (P2–P7 and P7–P14) and adolescent (P14–P21 and P21–P30). Tamoxifen was injected intraperitoneally twice with an interval of 24 h, and the utricle was removed at specific time points (Figure 4I). By counting cells co‐labelled with Sox9tdTomato and Myo7a, the efficiency of Sox9+ cell conversion into HCs was determined in all groups. In the first week, about one third of the HCs were derived from Sox9+ cells (Figure 4J,K,O, Videos 1 and 2). Between P7 and P14, the number of Myo7a+/Sox9tdTomato+ cells decreased (Figure 4L,O, Video 3), and newly generated HCs in the first 2 weeks accounted for around half of the HCs during this period. At the third and fourth weeks after birth, the percentage of HCs generated through differentiation continued to decrease (Figure 4M–O, Video 4). These results suggested that the capacity of Sox9+ cells to differentiate into HCs decreases dramatically in the second postnatal week and then maintains a relatively constant level from 3 weeks onwards.

**FIGURE 5:**
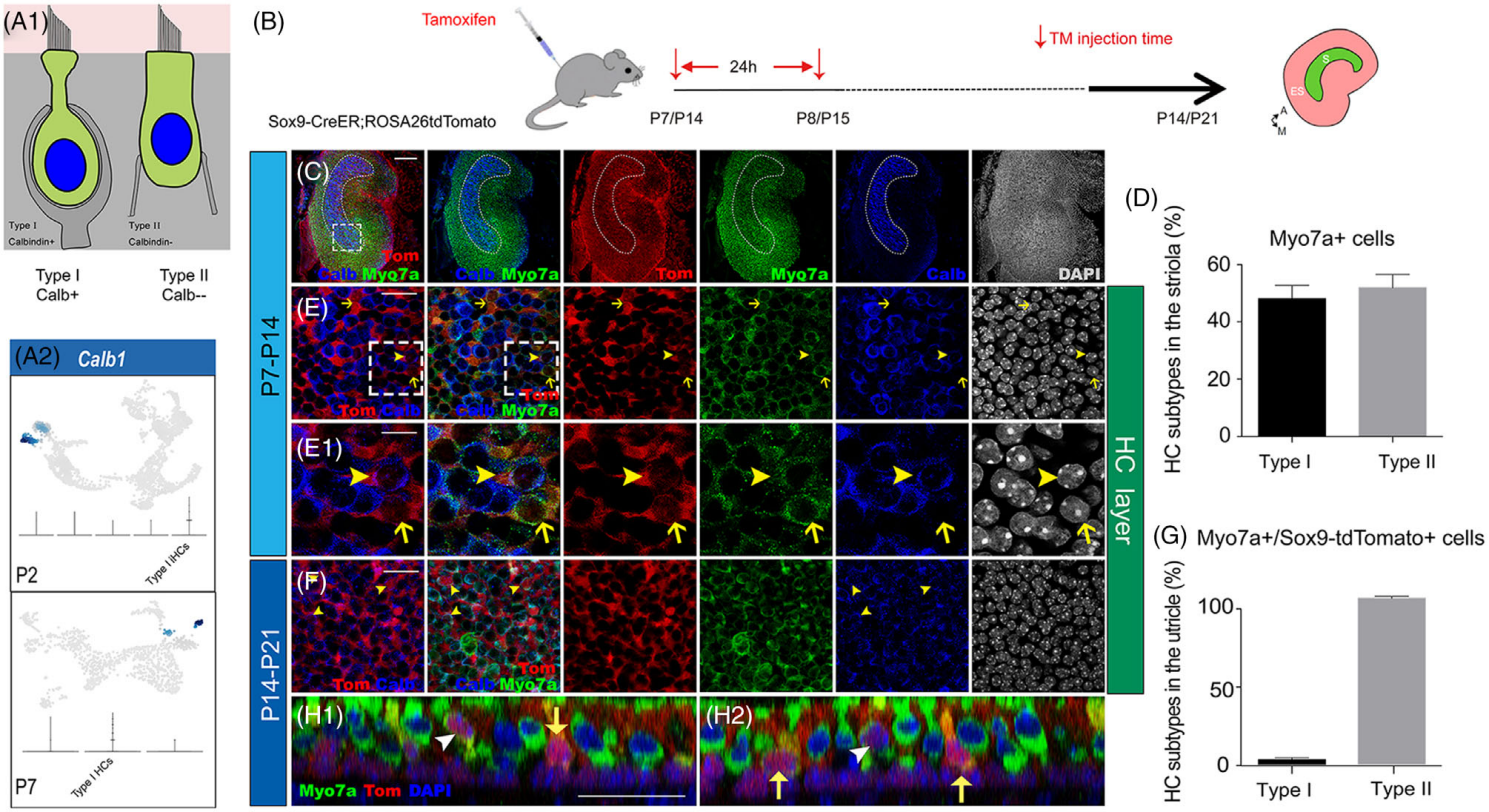
Cell fate of Sox9+ cells in HC differentiation. (A1) Diagram depicting the morphological characteristics and antigen specificities of Type I and Type II HCs. (A2) Density plots created by the Nebulosa package showing the RNA expression of Calb1 at P2 and P7. (B) Schematic describing the procedure of Sox9+ cell tracing. (C) Immunofluorescence assays in the mouse utricle after tamoxifen administration. The vast majority of calbindin+ cells were detected in the C‐shaped central region of the mouse utricle. (D) The proportions of Type I and Type II HCs in striolar HCs. (EE1) In the striola, a proportion of tdTomato+/Myo7a+ HCs showed immunoreactivity for calbindin (arrowheads), while other tdTomato+/Myo7a+ HCs were calbindin− (arrows). (F) Calbindin+/tdTomato+ HCs were only observable within 3 weeks after birth (arrowheads). (G) Proportions of new Type I and Type II HCs among the total new HCs. (H1,H2) The surface view of the Sox9tdTomato mouse utricle after tamoxifen treatment showed that both Type I HCs and Type II HCs participate in HC generation. Data are shown as the mean ± SD, *n* = 3; scale bars are 100 µm in C, 20 µm in E, F, and F1, and 10 µm in E1

Calbindin is a marker of Type I vestibular HCs in mammals^2^, ^40^, ^41^ (Figure 5A1). In P2 and P7 utricles, only a few immature Type I HCs expressed *Calb1*, and the expression level was relatively low (Figure 5A2). P7 and P14 Sox9tdTomato transgenic mice were used to identify the source of HCs (Figure 5B). Most of the calbindin+ cells were concentrated in a C‐shaped central region, which was in accordance with the location of Type I HCs as previously reported^42^ (Figure 5C). We found that half of the HCs in the striola were Type I HCs (Figure 5D) and that 3.07% ± 0.55% of the Myo7a+/Sox9tdTomato+ cells were labelled with calbindin (Figure 5E‐E1, F, G). The newly generated calbindin+/Myo7a+ cells were only observed within the first 3 weeks after birth. In addition, the morphological evidence indicated that both Type I and Type II HCs were turned over during this period, while most of the newly generated cells were calbindin− HCs (Figure 5H1,2).

### 2.6 Mitotic cells in the utricles

**FIGURE 6:**
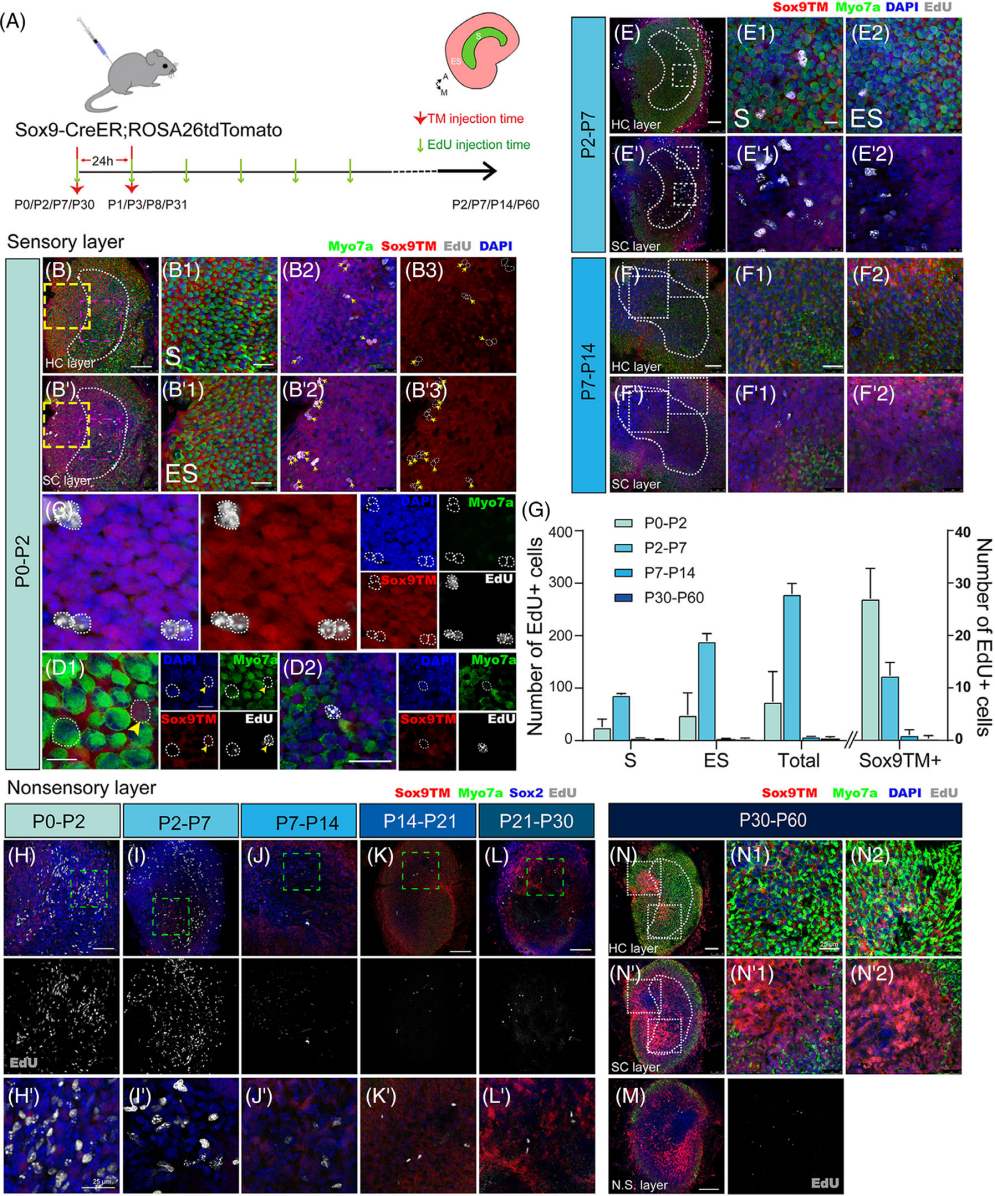
Mitotic proliferation capacity of Sox9+ progenitor cells. (A) Diagram describing the procedure of tamoxifen and EdU administration in Sox9tdTomato mice. (B–D) Laser confocal scanning images of utricles from Sox9‐CreER;tdTomato mice at P0–P2, focused on either the HC layer (B) or the SC layer (b). (B1–B3) Magnification of the approximate striolar region in Figure B and b (purple dotted rectangle). (B′1–B′3) Magnification of the approximate extrastriolar region in Figure B and b (yellow dotted rectangle). Large numbers of proliferating progenitor cells were detected in both the striola (B1–B3) and the extrastriola (B′1–B′3). (C) EdU+ cells are presented in pairs. (D1,D2) During the neonatal period, Myo7a+/Sox9tdTomato+/EdU+ cells and Myo7a+/Sox9tdTomato–/EdU+ cells were both detectable in neonatal mouse utricles at 48 h after tamoxifen treatment. Yellow arrowheads indicate Myo7a+/ Sox9tdTomato+/EdU+ cells. The fluorescent signal became weak or disappeared as progenitor cells proliferated. (C, D2). (E,E′; F,F′) Proliferating cells were captured in the P2–P7 and P7–P14 age groups in both the sensory epithelium (E–E2; F–F2) and the non‐sensory epithelium (E′–E′2; F′–F′2). (G) Left: Quantification and comparison of the numbers of EdU+ cells in different regions of the sensory epithelium. Right: Quantification of Sox9tdTomato+/EdU+ cells. (H–M) Numerous EdU+ nuclei were seen in the connective tissue underlying the sensory epithelium. (H,H′) P0–P2, (I,I′) P2–P7; (J,J′) P7–P14; (K) P14–P21; (L) P21–P30; and (M) P30–P60. (N–N2; N′–N′2) No EdU+ proliferative cells were detected in either the HC layer or the SC layer of adult mouse utricles. Scale bars are 100 µm in E, K, L, M and N; 50 µm in B, F and H; 25 µm in B1, B′1, F1, H′ and N1; and 10 µm in D1 and D2. Data are shown as the mean ± SD

We next investigated the proliferative ability of Sox9+ ENHCs. EdU was subcutaneously injected into the mice along with tamoxifen so as to label proliferating cells (Figure 6A). During the neonatal period, the number of EdU+/Sox9TM+/Myo7a‐ cells detected per utricle was 80.375 ± 5.121(*n* = 4) (Figure 6B‐B3, B′‐B′3), and over 80% of the proliferating cells appeared in pairs (Figure 6C). Myo7a+/EdU+/Sox9tdTomato+ and Myo7a+/EdU+/ Sox9tdTomato− cells appeared in both the striola and extrastriola within 48 h after tamoxifen administration at P0 (Figure 6D1,2). Additionally, we did not observe any Myo7a+/Sox9tdTomato+/EdU+ cells at other stages.

ENHCs that proliferated and completed mitosis could also be captured in the juvenile groups (Figure 6E,F); however, the proliferation efficiency drastically declined with age (Figure 6G). Few EdU+ cells were observed in the sensory epithelium in the adolescent and adult groups (Figure 6H–M). In addition, a very small number of EdU+ cells could also be found during this period from P30 to P60 (Figure 6N‐N′). These results indicated that Sox9+ cells only make a substantial contribution to the growth of the mouse utricular HCs through proliferation at the relative early stage of development. However, in both juveniles and adults, the Sox9+ ENHCs were mainly involved in HC differentiation. Thus, the question lies in what is the main source of mitotically generated HCs after birth.

**FIGURE 7:**
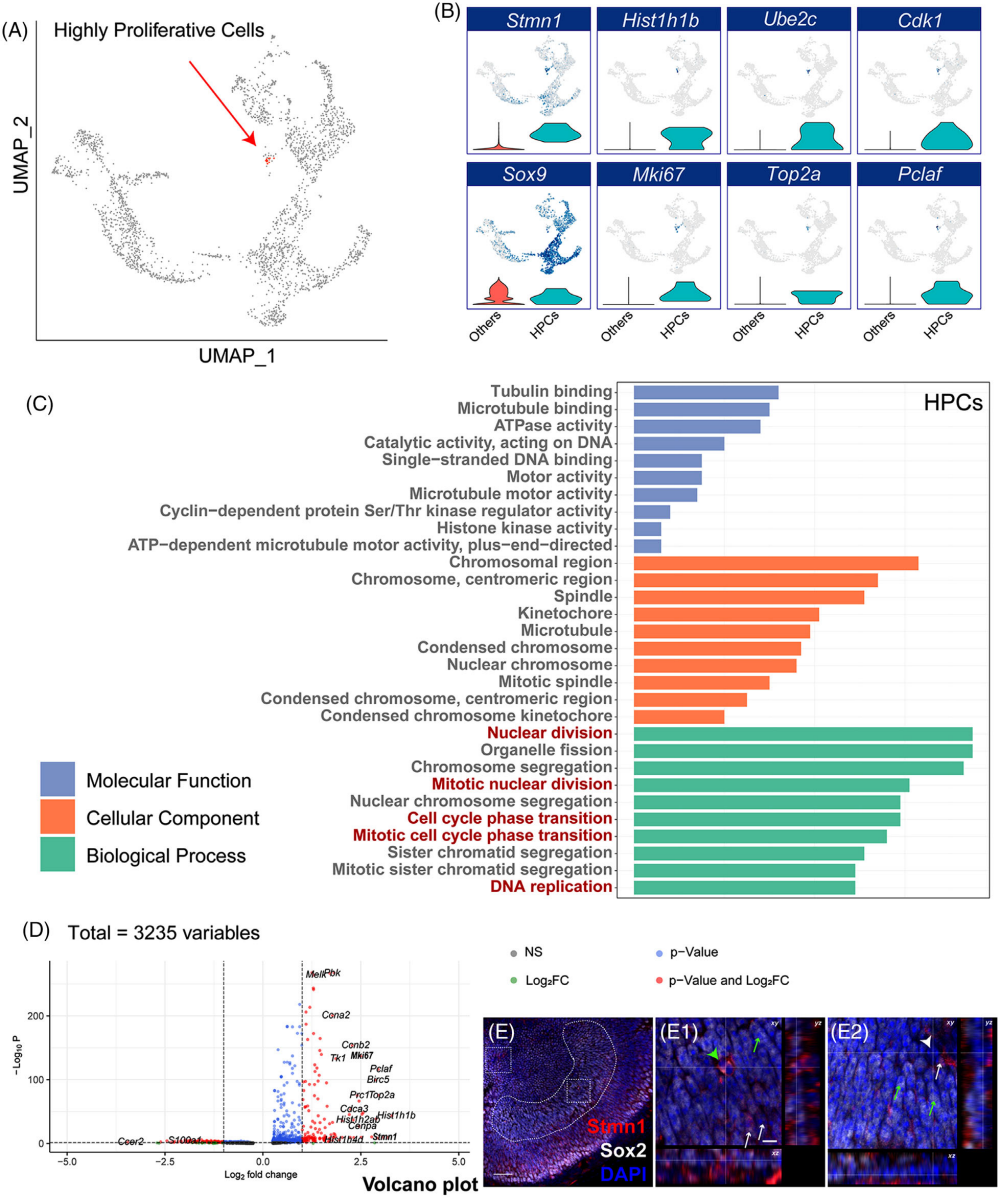
Potential mitotic generation of utricle sensory epithelium cells. (A) UMAP clustering of HCs, progenitors, and precursors from P2 mouse utricle sensory epithelium. (B) The marker genes of highly proliferative cells visualized with feature plots and violin plots. (C) Gene ontology (GO) analysis of genes upregulated in highly proliferative cells. The three sub‐ontologies (Biological Process, Molecular Function and Cellular Component) of the GO hierarchy are shown by different colours. GO items are in order of enriched gene numbers. (D) Volcano plot describing the differentially expressed genes between highly proliferative cells and other cells. The Log2(fold‐change) cut‐off was set to 2, and the *p*‐value cut‐off was set to 0.001. (E) Immunofluorescence labelling of Stmn1 and Sox2. (E1,E2) Magnification of the approximate striolar region (E1) and extrastriolar region (E2) in Figure E (white dotted rectangle). The light green arrowhead indicates cells with high expression of Stmn1, which suggests that these might be proliferative cells. The white arrowhead indicates a Sox2−/Stmn1− cell, and the light green arrows indicate Sox2+/Stmn1− cells. The white arrows indicate Stmn1+/Sox2− cells. Scale bars are 50 µm in E and 10 µm in E1

We allocated the proliferative cells according to cell cycle marker genes by scRNA‐seq analysis. It is notable that there was a highly proliferative cell population identified through UMAP dimensionality reduction (Figure 7A). These cells showed high RNA expression levels of *Ube2c*, *Cdk1*, *Top2a*, *Hist1h1b*, *Mki67* and so forth (Figure 7B), which were found to be related to increased cell division and cell cycle phase transition according to GO analysis (Figure 7C). This cell subtype could be distinguished by several markers (Figure 7D) such as *Stmn1*, which was expressed in the striolar SCs as shown by the immunostaining signals in Figure 7E. The highly proliferative cells could also be found in P7 mice (Figure S6), but these cells could not be detected in P30 mice by scRNA‐seq.

**FIGURE 8:**
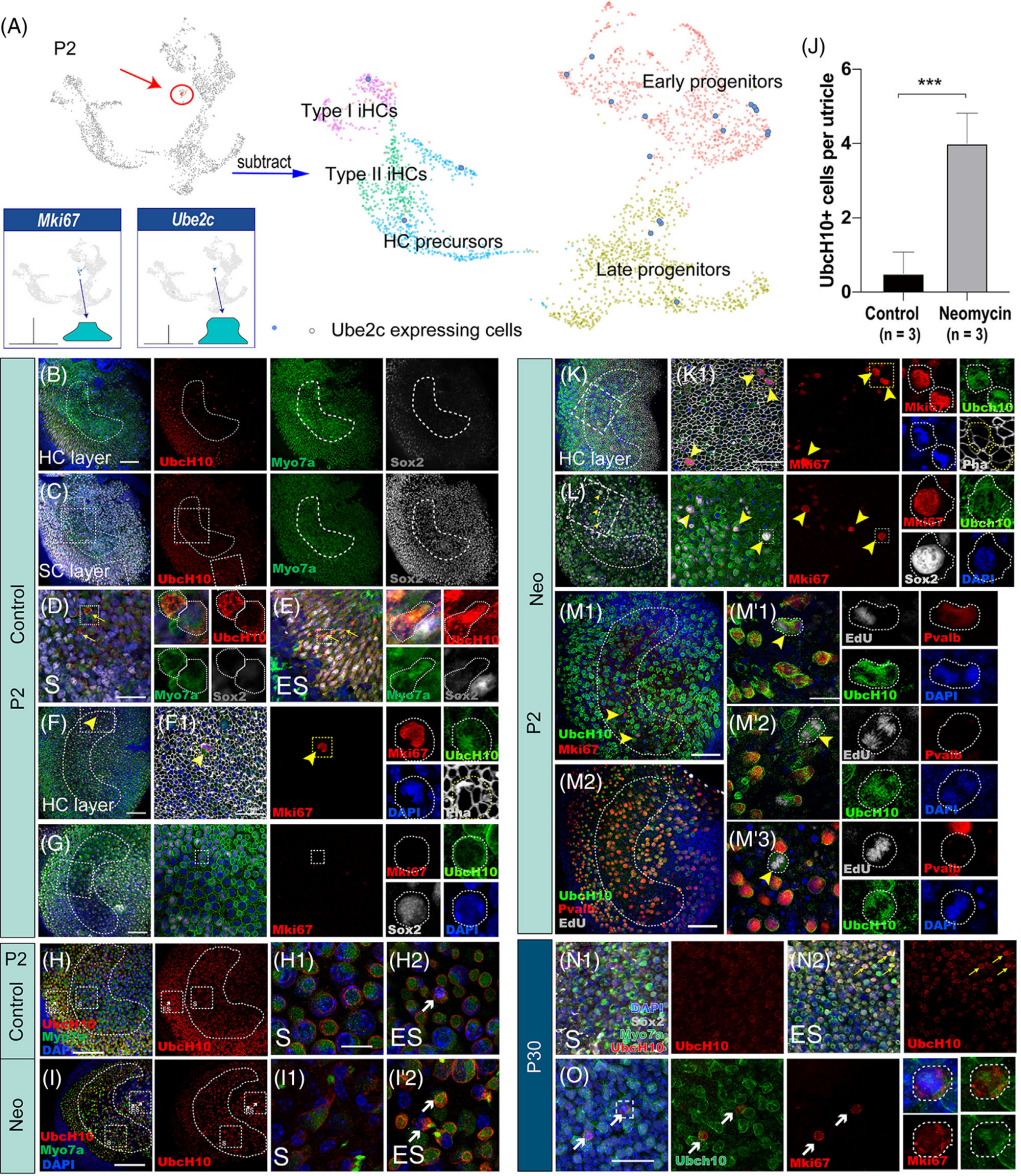
Highly proliferative cells were detected in the utricle. (A) Most of the Ube2c+ cells could be sorted into progenitors when cell cycle genes were regressed in the Ube2c+ subpopulation. (B,C) Laser confocal scanning of the HC layer (B) and the SC layer (C) after co‐labelling with Myo7a, UbcH10 and Sox2 in P2 mouse utricles. (D,E) Magnified images in the striola (D) and extrastriola (E) of the P2 mouse utricle. The yellow arrows indicate UbcH10 expression in the cytoplasm of Myo7a+ cells, while in other cells UbcH10 immunoreactivity was always found on the cell membrane. The panels on the left of D and E show the enlarged views of UbcH10+ cells. (F,G) Cells expressing UbcH10 in the cytoplasm were also labelled by Mki‐67 (F), while cells expressing UbcH10 on the cell membrane were not. The yellow arrowheads indicate MKi67+/UbcH10+ double‐positive cells (G). (H‐H2,;I‐I2) Laser confocal immunofluorescence images of Myo7a and UbcH10 in neomycin‐damaged utricles in neonatal mice compared to the control samples. The white arrows indicate Myo7a and UbcH10 co‐labelled cells in the control group (H1,H2) and the neomycin‐treated group (I1, I2) in vitro. (J) Quantification and comparison of the numbers of UbcH10+ cells in the utricles between control and neomycin groups (*** *p* < .001 vs. control). (K,L) After damage, MKi67+/UbcH10+ double‐positive cells were mainly concentrated in the HC layer (K) and expressed Sox2 (L). (M1–M′3) UbcH10+ cells were co‐labelled with EdU, and these cells expressed parvalbumin (M′1), did not express parvalbumin (M′2), or showed punctate expression of parvalbumin (M′3). The yellow arrowheads indicate EdU+/UbcH10+ double‐positive cells. (N1,N2) Magnified images in the striola (N1) and extrastriola (N2) of the P30 mouse utricle. The yellow arrows indicate UbcH10 expression in the cytoplasm of Myo7a+ cells. (O) Immunofluorescence staining showing the existence of Mki‐67+/UbcH10+ co‐labelled cells in the extrastriola of P30 utricles. The white arrows indicate MKi67+/UbcH10+ double‐positive cells. Data are shown as the mean ± SD, *n* = 3; scale bars are 100 µm in B and C; 50 µm in F, G, H, I, K, L, M1 and M2; 20 µm in D, F1, K1, N1 and O; and 10 µm in H1, I1 and M′. Data are shown as the mean ± SD. Neo: neomycin

We next verified the expression of UbcH10 (the protein encoded by *Ube2c*) in mouse vestibular ENHCs. UbcH10 is a member of the anaphase promoting complex/cyclosome (APC/C), and it promotes the degradation of several target proteins during cell cycle progression, particularly mitotic cyclins during the metaphase/anaphase transition.^43^ Interestingly, when we subtracted cell cycle genes from all of the cells, the UMAP analysis indicated that most of the *Ube2c*+ cells could be sorted into progenitors, with very few *Ube2c*+ cells seen among the HCs (Figure 8A), which indicated that these cells could be regarded as proliferative progenitors or HC precursors.

We used immunohistochemistry to validate the *Ube2c* expression and found that UbcH10 was broadly expressed on the cell membranes (Figure 8B–E), and only a small number of cells in both the striola and extrastriola were UbcH10+ in the cytoplasm (Figure 8D,E, yellow arrows). It has been reported that *Ube2c* is located in the nucleus and cytoplasm^44^, ^45^ and that its expression is regulated during the cell cycle. The level of *Ube2c* is low in G1 phase, gradually accumulates in S and G2 phases, reaches its peak at mitosis, and then decreases sharply as cells exit from mitosis.^46^, ^47^ According to the scRNA‐seq data, *Ube2c* was highly expressed in only a very small number of cells. Therefore, we hypothesized that the cells that expressed UbcH10 in the cytoplasm should be highly proliferative and functional cells. To test this hypothesis, we performed co‐labelling of UbcH10 with Mki67, a proliferation marker. The results showed that cells expressing UbcH10 on the cell membrane did not co‐label with Mki67 (Figure 8G), but cells undergoing high levels of mitosis expressed Ki67 and UbcH10 in the cytoplasm (the yellow arrowheads, Figure 8F).

To further understand the role of UbcH10+ in HC regeneration, we also examined UbcH10 expression in neomycin‐damaged utricles in neonatal mice. Neomycin at a concentration of 2 mM was added to the medium for 24 h, and the explants were collected 48 h later. Compared to the control sample, there were many more UbcH10+ cells (the white arrows in Figure 8H,I, Figure 8J) in the neomycin group, and these were distributed mainly in the extrastriolar region (Figure 8H,I). The UbcH10+ cells could be co‐labelled with cell proliferation markers such as Mki67 or EdU (Figure 8K–M). Interestingly, the UbcH10+ cells in the neomycin‐damaged specimens were also parvalbumin+ (Figure 8M′1), which indicated that HCs might proliferate from progenitor cells directly after damage.

In addition, we observed some Mki‐67+/UbcH10+ cells in the extrastriola in the P30 WT mouse utricle that were co‐localized with either Myo7a or Sox2 (Figure 8N1–N2,O), which again suggested that the proliferative cells were UbcH10+ cells in adult mice. This observation further demonstrated the expression profile characteristics of proliferative cells. Taken together, these results indicated that in adult mouse utricles there are still a few new HCs that appear to arise from mitotic generation in the extrastriola.

## 3 DISCUSSION

Here we report evidence for the heterogeneity of utricular ENHCs in the inner ear, including progenitor cells in newborn mice and SCs in adult mice, by scRNA‐seq analysis.

### 3.1 scRNA‐seq is a powerful tool in utricular cell profile analysis

scRNA‐seq is a powerful method for showing cellular diversity in the utricular sensory epithelium. Benefiting from continuous improvements in the digestion procedure, we were able to generate an adequate amount of high‐quality single‐cell suspension for creating the sequencing library. The single‐cell transcriptomes of over 10 000 sensory epithelial cells from P2, P7 and P30 mouse utricles were determined. We thus expand upon previous findings in the mouse utricle^48^ because the 10X Genomics Chromium approach implemented here has a relatively higher cell throughput and can detect rare cell clusters more accurately.^49^

ENHCs, including progenitor cells and SCs, might be useful in the development, function and regeneration of other inner ear cell types,^50^ particularly in the recovery process after HC damage. Based on the hypothesis that ENHC subsets might have different potentials regarding distinct biological processes, the heterogeneity of inner ear ENHCs has attracted increasing attention. Because ENHCs only displayed limited ability to transform into HCs, a better understanding of ENHC heterogeneity might lay the foundation for improving regeneration efficiency. Earlier results mainly reflected SC heterogeneity through morphological observation,^51^ but only minor morphological differences were observed in the SCs of the utricular sensory epithelium.^52^ The 10X scRNA‐seq system offers similar detection rates for various forms of sensory epithelial cells, and its relatively higher sensitivity makes it a better choice for cell clustering. More importantly, 10X scRNA‐seq technology detects cell heterogeneity at the RNA level, thus providing more comprehensive results compared with other molecular methods. Also, because 10X technology allows the detection of lowly expressed transcripts, we were able to detect rare cell clusters, including the highly proliferative cell subset in P2 sensory epithelial cells. The proliferative cell cluster had relatively higher *Stmn1*, *Ube2c*, *Cdk1* and *Mki67* RNA expression levels. When cell cycle genes were regressed, the highly proliferative Ube2c+ cells could be classified into both ENHC and HC subtypes. These results indicate the potential occurrence of cell mitosis in the utricle sensory epithelium. Macrophage subsets that could migrate and locate into the non‐sensory epithelium of the utricle could also be detected.^53^

Since the discovery of the direct transformation process of SCs into HCs,^54^ many studies have been undertaken to seek an effective strategy to replenish lost HCs from the SC pool. However, cell fate changes in SCs in the mature utricle sensory epithelium hinder the process because SCs lose their plasticity with age. Elliott et al. (2021) investigated the effects of the loss of *Bdnf*, which leads to the significant loss of HCs at the periphery in very old mice after loss of vestibular neurons. The vestibular HC loss in mice might be due to the gradually lost connections with vestibular ganglion neurons over the first 6 months of life.^55^ Previous studies have tended to attribute the loss of SC plasticity to cytoskeletal changes. Cross‐species SC comparisons have shown that SC regenerative ability is associated with intercellular junctions,^56^, ^57^ and there is no denying that cytoskeletal changes affect the cell's ability to alter its morphology, which hinders postnatal SC proliferation. Based on the scRNA‐seq data, we implemented tools for quantitatively inferring intercellular communication networks and compared the relative information flow between SCs and HCs in the sensory epithelium at different time points, thus providing deeper insight into the carefully regulated process of cell fate switching.

Regarding the study of vestibular sensory organ development, the fundamental goal is to map the developmental history of each individual cell in terms of localization, composition and major functions. With the rapid development of single‐cell transcriptome analysis, powerful methods for inferring state trajectories can be used to comprehensively describe the heterogeneity of cell differentiation at the cell‐cluster level and to provide information regarding the pseudotime dimension, which measures the progress of the transition process. Nevertheless, compared with cell state tracking measurement through lineage tracing, which is considered the gold standard, sequencing of states based on scRNA‐seq data cannot correlate progenitor cells with future cell states as effectively due to the fact that the implementation of unsupervised learning algorithms in the trajectory analysis might produce misleading results. Also, the single‐cell trajectory landscape will inevitably miss certain cellular properties due to time gaps in sample acquisition and hidden variables due to the random sequencing process and to the limitations of different sequencing methods. In this study, we used Sox9tdTomato mice as reporters for lineage tracing of vestibular ENHCs. By combining lineage tracing results with scRNA transcriptomes at different timepoints, we could detect potential progenitor cell pools at P2 and thus provide a more thorough description of the origin of newborn HCs at P30. These observations were further verified through Cre‐LoxP lineage tracing. Thus, in our study perspective lineage tracing and scRNA‐seq complemented each other to reach full coverage of the entire developmental timeline and of the various cell types.

### 3.2 Subtypes and properties of utricular ENHCs

Mammalian vestibular SCs are quite different from auditory SCs. In adult animals, the cochlear SCs can be separated into at least eight distinct subtypes,^30^, ^58^, ^59^ but vestibular SCs are seemed not yet specialized even in adulthood. Vestibular SCs are thought to be relatively morphologically homogeneous,^52^ but recent evidence suggests that there are distinct markers of vestibular SCs. Stone et al.^60^ reported four CreER lines of transgenic mice that have different expression patterns, which further suggests that utricular SCs might be heterogeneous. Recently, researchers spatially divided vestibular SC subtypes into striolar and extrastriolar subgroups based on their unique transcriptomes.^48^ Studies have also shown that striolar SCs express otolithic membrane glycoproteins and other markers,^13^ and when HCs are damaged the Wnt target gene *Lgr5* can be activated in striolar SCs in neonatal mouse utricles.^61^ Furthermore, previous studies analysed the profiles of both Plp1+ and Lgr5+ SCs, which are expressed in the striola and extrastriola, respectively.^12^ These studies have therefore indicated that there are likely to be more than two subtypes of SCs in the utricles.

The other property of utricular SCs in adult mammals that is different from cochlear SCs is their regenerative capacity. By labelling SCs with mitotic cell markers, a previous in vivo study failed to find any tritiated thymidine‐labelled HCs in the vestibular sensory epithelium even though they found immature HCs after ototoxic damage.^54^ However, proliferating SCs could be labelled. Interestingly, many labelled stromal cells, pericytes and Schwann cells were observed in the tissues underlying and adjacent to the sensory epithelium, which suggests that the stroma might be a highly proliferative tissue. Such a conclusion is supported by recent scRNA‐seq experiments.^48^

In the present work, we have identified three subtypes of ENHCs in the neonatal utricles by scRNA‐seq, including two subtypes of progenitor cells and one subtype of HC precursor cells. Similar to the findings of Burns et al.,^13^ we found that the early progenitors shared similar characteristics with the translational epithelial cells (Figure S7). However, the number of cells we analysed was about 17 times greater than the number used in the previous studies, and therefore we could perform pseudotime analysis and could draw the internal trajectory tracks of ENHC development and differentiation. We further identified trajectories between subtypes of cells at P7; however, we failed to find trajectory tracks between the subgroups in the adult mice, which indicated that in P30 utricles most cells are maintained in a balanced and stable state. Therefore, the ENHCs in P30 utricle can be defined as mature SCs.

The mature SCs are heterogeneous, and they could be classified into six clusters with different functions at P30. A small population of *Tectb*+ cells were located in the striola, which indicated cells that derived from *stem cell differentiation* (Figure 3D). Another subtype of cells might be involved in gliogenesis or gland morphogenesis and thus might be the source of otolith formation. Moreover, some cells were highly metabolic and participated in oxidative phosphorylation. Together, these findings strongly support the hypothesis that utricular ENHCs are heterogeneous.

We found that the ENHCs in neonatal utricles were mainly composed of progenitor cells, which are totally different from adult ENHCs. First, the cell crosstalk and communication are much more comprehensive in neonatal utricles. The signalling pathways, including Tenascin, MDK, CDH, Notch and WNT, were only found in the neonatal stage, which is in accordance with previous studies. Second, because cell fates of most neonatal ENHCs were not determined, these cells could be classified into progenitors or HC precursors according to the pseudotime analysis. Among early progenitors, there were some highly proliferative cells marked by both *Ube2c* and *Mki67*. After we subtracted the cell cycle genes, the *Ube2c+* cells could be classified into other progenitors or HC subtypes, which indicated that their transcriptomes were similar to other ENHCs or HCs except for the expression of cell cycle genes. Third, *Sox9* is an important gene in the developing utricle and was expressed in almost all of the ENHCs in the neonatal period. As previously reported, in the embryonic stage *Sox9* and *Sox2* expression overlap in most prosensory and sensory domains, and Sox9 is ultimately restricted to SCs.^39^ In the present study, we found that Sox9 was expressed in ENHCs at P2, P7 and P30. Sox9+ cells give rise to new HCs at different ages, but the progenitor cells that specifically transdifferentiate into hair cells need to be further verified. Notably, Sox9 was also expressed in the non‐sensory domain. Finally, the expression of *Gata2*, another marker gene of progenitors in early development in mouse embryos, is predominantly detected in the dorsal vestibular system and is required from E14.5–E15.5 onward for proper vestibular morphogenesis.^37^, ^62^ In contrast to *Gata2*, *Gata3* is most prominently expressed in the cochlear duct and the nearby periotic mesenchyme during inner ear development.^62^ The expression of *Gata3* is weak in the dorsal vestibular region, and it is uniquely expressed in the striola region in the developing utricle.^63^ In the present study, we confirmed that Gata2 was mainly located in the sensory epithelium of the utricle and that its expression decreased over time. The decrease in Gata2 was in accordance with the proliferative capacity of utricular cells. Interestingly, we also found some Gata2+ neural fibres in the stromal layer, which gradually disappeared in adulthood. These Gata2+ fibres were not co‐localized with parvalbumin.

### 3.3 Utricular HCs are mainly differentiated from Sox9+ ENHCs

Spontaneous HC regeneration in the utricle included SC differentiation and mitotic regeneration, and we found that these HCs originated from different subtypes of ENHCs, among which Sox9+ cells could directly differentiate into HCs.

Sox9 is a transcription factor containing the highly conserved SRY‐related high mobility group DNA binding domain, and it is a critical regulator during development.^64^ Sox9 is expressed in ENHCs but not in HCs starting from E18.5 in the utricular sensory epithelium,^39^, ^65^ and anti‐Sox9 antibodies specifically label SC nuclei with no significant labelling of HCs.^60^ This cell type‐specific expression persists throughout postnatal life in both the striola and extrastriola.

In this study, Sox9‐CreERT;ROSA26‐tdTomato mice were used as reporters for lineage tracing of vestibular ENHCs. At 48 h after tamoxifen injection, most of the ENHCs were Sox9tdTomato+ in both the striola and extrastriola. Furthermore, 4.02% ± 0.52% of the total HCs were Sox9tdTomato+, and at 72 h after injection 7.68% ± 0.20% of the HCs were Sox9tdTomato+. These labelled HCs might reflect ENHCs converting into HCs over a very short time period. To determine the origin or the progenitor cells of these newly regenerated HCs, Cre recombinase was activated in the adult mice. Although the percentage of Myo7a+/Sox9tdTomato+ cells decreased, we could not exclude efficient recombinase activity in a small number of HCs in the Sox9CreERT2 mice. Stone^60^ compared and analysed the activity of Cre recombinases from 11 transgenic mice in the adult mouse utricle, confirming that the Cre enzyme from Sox9CreERT2 mice was activated in all SCs and that red fluorescent protein was expressed in only a small number of HCs. Thus, we conclude that the Sox9tdTomato mouse is a powerful tool for marking almost all SCs.

### 3.4 Proliferative HC progenitor identification in the mouse utricle

Interestingly, we found some highly proliferative cells marked with UbcH10 in the adult utricles, which suggests that even in the intact utricles there are still progenitors that should be the source for new proliferative utricular HCs, and these progenitor cells were mainly located in the extrastriola during adulthood.

In non‐mammalian vertebrates, vestibular HCs are generated throughout the animal's life.^16^, ^26^ In mice, spontaneous vestibular HC generation predominantly occurs during gestation and in the neonatal period,^24^, ^25^ and it appears that HC accumulation begins in the periphery. During development, the addition of utricular HCs in rodents occurs in a central‐to‐peripheral gradient,^25^ and during adulthood the newly generated HCs might migrate toward the centre of the utricle epithelium over time. However, immature and newly generated HCs can be detected in the adult utricle.^26^ Although a previous publication reported the discovery of pluripotent stem cells in the adult mouse inner ear,^66^ there has been no direct evidence to trace or mark such cells.

To determine whether the newly generated HCs in the present study were produced from mitotic cell divisions, EdU was injected at different ages. Both EdU+ HCs and EdU+ ENHCs were detected in pairs, confirming the proliferative generation of HCs in the postnatal stage. Although few EdU+ ENHCs were observed in the sensory epithelium 2 weeks later, we could still mark the proliferative cells with Mki67 in P30 mice. We note that the triple marked cells (Myo7a+/EdU+/Sox9tdTomato+) could only be detected at P0–P2. Meanwhile, the number of EdU+/Sox9tdTomato+ cells decreased dramatically from P2, which indicated that sensory cells lost most of their proliferative ability in the postnatal stage. A possible explanation for this is that Sox9+ cells derived from highly proliferative cells and then differentiated into HCs by chance. Due to the half‐life of the EdU and tamoxifen solution, not all of the proliferating cells could be captured at later stages.

Cell cycle progression and differentiation may be closely related to ubiquitination‐dependent proteolysis.^67^ UbcH10, also referred to as ubiquitin conjugating enzyme E2(UBE2C), is a member of the anaphase promoting complex/cyclosome (APC/C), and it promotes the degradation of several target proteins during cell cycle progression, particularly mitotic cyclins during the metaphase/anaphase transition.^68^ UbcH10 is APC/C specific and is involved in progression through mitosis. UbcH10 is weakly expressed in many of the normal tissues but is highly expressed in different malignant human cancers; however, its function in the auditory system has rarely been studied. In the present work, we observed that some of the newly generated HCs expressed Mki67 and UbcH10, which indicates that they might arise via mitotic regeneration. An alternative explanation for the observed phenotype could be that in response to DNA damage the existing parvalbumin+ HCs incorporate EdU and UbcH10 expression is induced. Our results thus provide direct evidence for HC generation through mitotic cell proliferation and subsequent differentiation. Furthermore, we used the novel proliferative marker UbcH10 to label the mitotic progenitors. UbcH10+ cells lost their stemness with age (Figure S8), and we think that this subpopulation of cells might disappear at some timepoint and that most newly generated HCs after that time might arise through the direct differentiation of progenitor cells. The role of UbcH10, which is expressed only in the cytoplasm of proliferative cells or progenitor cells, needs to be further investigated. It was also notable that UbcH10 could be detected on the membranes of most cells; however, the mechanisms behind this phenomenon are not known.

In addition, it is very difficult to detect UbcH10+ cells in the adult mouse inner ear. This might be due to the protein level decreasing with age. It is also difficult to identify the Mki67+ cells in adults. We only detected a very small number of Mki67+/UbcH10+ cells in some of the mice because they only appeared over a very short period and were very hard to capture.

## 4 CONCLUSION

ENHCs in the utricle are heterogeneous in both neonatal and adult mice. Sox9 is dynamically expressed in developing utricular cells but is ultimately constrained to only being expressed in ENHCs in adult mice, and these Sox9+ ENHCs might be a source of directly differentiated HCs. There are still a few HCs generated by mitotic proliferation in adults, which mainly occurs in the extrastriola. UbcH10 might therefore be a proliferative HC progenitor marker that can be detected during both the neonatal period and in adulthood. Together, these findings expand our understanding of ENHC characteristics and HC generation in the adult vestibular organs in mammals.

## 5 MATERIALS AND METHODS

### 5.1 Mouse models and drug administration

Wild‐type C57BL/6J mice of different ages were purchased from Jiesijie Experimental Animals Co., Ltd. (Shanghai, China). ROSA26R‐tdTomato (Cat. #7908) mice in the C57BL/6J background were purchased from the Jackson Laboratory. Sox9‐CreERT2^69^ mice were kind gifts from the Shanghai Academy of Life Sciences. For activating the Cre recombinase, tamoxifen (Sigma‐Aldrich) diluted in corn oil was administered by subcutaneous or intraperitoneal injection at different time points. Each injection was 0.20 mg/g bodyweight once on two consecutive days. To label proliferating cells and their progeny, the thymidine analogue 5‐ethynyl‐2**′**‐deoxyuridine (EdU) (Click‐iT EdU Imaging Kit; Invitrogen) was injected at 50 mg/kg body weight intraperitoneally once a day according to the experimental requirements. All animal procedures were approved by the Animal Care and Use Committee of Fudan University and were consistent with the National Institutes of Health Guide for the Care and Use of Laboratory Animals.

### 5.2 Tissue samples utricle explant culture

Mice at different ages were sacrificed by cervical dislocation, and the temporal bones were removed and submerged in 4% paraformaldehyde for 2 h at room temperature or overnight at 4°C. After fixation, the temporal bones were stored in PBS and whole utricles were dissected out of the temporal bones and transferred into histochemical dishes for free‐floating immunofluorescent labelling. The otoconial membranes were removed with fine forceps, and the otoconia was removed by soaking the tissue in 10% EDTA at room temperature for 10 min.

P2 mice were used in the explant culture. The euthanized mice were sacrificed and the utricles were isolated in tissue culture medium under sterile conditions. The otoconia in the utricles were gently removed with fine forceps. Whole organs were cultured in DMEM/F12 (Invitrogen) supplemented with 1% N2 (Invitrogen), 2% B27 (Invitrogen) and ampicillin (50 mg/ml; Sigma‐Aldrich) at 37°C with 5% CO2 in 4‐well Petri dishes (Greiner Bioone). Neomycin sulphate (2 mM, Sigma‐Aldrich) was added to the medium at a concentration of 2 mM for 24 h to kill the HCs, and the explants were collected 48 h after withdrawal of neomycin.

### 5.3 Immunohistochemistry

After fixation, the tissues were incubated in blocking buffer (10% donkey serum in 10 mM PBS with 1% Triton X‐100) for 1 h at 37°C and incubated with primary antibody overnight at 4°C. The primary antibodies are listed in Table S1. After rinsing three times with 10 mM PBS, the utricles were incubated with the appropriate secondary Alexa Fluor‐conjugated antibodies for 1‐2 h at room temperature on the following day. HC bundles were labelled with phalloidin (1:200 dilution; Life Technologies) and nuclei were labelled with DAPI (1:800 dilution; Sigma‐Aldrich). Proliferating cells were labelled with EdU (Click‐iT EdU Imaging kit, Life Technologies) according to the manufacturer's protocol.

### 5.4 Image acquisition, cell counts and nuclear diameter measurement

Fluorescent images were acquired using a Leica SP8 confocal microscope, and randomly chosen representative areas of the striola and extrastriola were captured for analysis. All of the images were digitally processed using ImageJ, Adobe Photoshop CC 2015 and Adobe Illustrator CC 2017. The numbers of Myo7a+ cells, Sox2+ cells, and tdTomato+/EdU+, Myo7a+/tdTomato+, and Myo7a+/tdTomato+/EdU+ co‐labelled cells were counted manually in the striola and extrastriola separately using the Cell Counter plug‐in in ImageJ. ImageJ was used to measure the diameters of the HCs and SCs.

### 5.5 Isolation of utricular cells for scRNA‐seq analysis

P2, P7 and P30 mice of both sexes were euthanized and their utricles were dissected (*n* = 20) in cold RNase‐free Dulbecco Phosphate Buffered Saline. Utricles of P2 and P7 mice were further transferred to DMEM/F‐12 with 0.2 mg/ml of thermolysin (Sigma, T7902) and 10 kunitz/ml of DNase I (Stem Cell Technologies, 07900) for 10 min at 37°C. After incubation, the utricular roof and stromal cells were dissected away to harvest the pure sensory epithelium. Utricular sensory epithelium from P2 and P7 mice and utricles from P30 mice were collected separately in 1.5 ml tubes and incubated in Accutase (Thermo Fisher Scientific, 00‐4555‐56) for 15‐20 min at 37°C with gentle trituration every 5 min (50 times with a 200 µl low‐retention pipette). The samples were then centrifuged at 300 × *g* for 5 min, and the dissociated cells were resuspended in 500 µl DMEM/F12 and filtered through a 40‐µm cell strainer. After centrifuging at 300 × *g* for 5 min, the cell pellets were resuspended with PBS. Cell viability was measured with trypan blue staining

### 5.6 scRNA‐seq analysis

scRNA‐seq was performed using the Illumina 10X Genomics system. We implemented the Chromium Single Cell 3′ v3 libraries under the guidance of the official instruction manual. Raw fastq files were aligned to the mouse genome (mm10, v2020‐A) and quantified using the CellRanger software package (v3.1.0(https://support.10xgenomics.com/single‐cell‐gene‐expression/software)), python (v2.7.14) and samtools (v1.7) with the default parameter settings. We captured 6 251 cells from the P2 mouse utricle (mean reads per cell = 44 444, median genes per cell = 1 396), 2,785 cells from the P7 mouse utricle (mean reads per cell = 1 17 233, median genes per cell = 2 387) and 8 918 cells from the P30 mouse utricle (mean reads per cell = 40 170, median genes per cell = 717). All further analyses based on CellRanger outputs were run using Seurat (R package v4.1.0, https://github.com/satijalioconrat) unless mentioned otherwise.

### 5.7 Processing of the scRNA‐seq datasets at the three time points

P30 cells with <200 unique genes or >7500 unique genes were excluded from the analysis, and cells with >35% mitochondrial genes present were excluded from downstream steps. After filtering, 8 781 P30 cells were included in the downstream analysis. P7 cells with <200 unique genes or >8000 unique genes and cells with >30% mitochondrial genes present were excluded from downstream steps. After filtering, 2 766 P7 cells were included in the downstream analysis. P2 cells with <200 unique genes or >8000 unique genes and cells with >30% mitochondrial genes present were excluded. After filtering, 6186 P2 cells were included in the downstream analysis.

Count matrices of all samples were processed using the SCTransform procedure with default parameters to perform a regularized negative binomial regression using 3 000 variable features. Cell cycle and ribosomal genes were filtered out. All datasets were processed under principal components analysis using the RunPCA functions in Seurat. The UMAP nonlinear dimensionality reduction method was implemented to generate two‐dimensional map coordinates. Cluster analysis was performed using the FindNeighbours and the FindClusters function (resolution set to 1.0).

Based on UMAP embedding, potential cells located in the sensory epithelium region were extracted from the whole dataset of P30, P7 and P2 cells using the FindAllMarkers function based on pre‐reported cell markers and the immunofluorescence staining results. A total of 7 758 P30 cells, 1 133 P7 cells and 2 737 P2 cells were included for the next steps in the analysis. The FindMarkers procedure in Seurat was used on each cluster to obtain differentially expressed genes for further annotations. Differentially expressed genes were also used for generating the cluster heat map depicting the marker genes of each SC cluster in P30 mice using ggplot2(R package v3.3.5(https://ggplot2.tidyverse.org/)) and ComplexHeatmap (R package v2.8.0(http://www.bioconductor.org/packages/release/bioc/html/ComplexHeatmap.html)).

### 5.8 Single cell dataset integration

The RunHarmony function in Harmony (R package v0.1.0(https://github.com/immunogenomics/harmony)) was used to remove the batch effects during the combination of P2 progenitors, P7 ENHCs and P30 SCs. Lambda was set to 0.8, calling for a stricter correction. Other parameters were set as defaults.

### 5.9 Functional enrichment analysis

Gene ontology (GO) functional enrichment analysis was performed with clusterProfiler (R package v4.1.4(https://bioconductor.org/packages/release/bioc/html/clusterProfiler.html)) and visualized using the ggplot2 R package. Org.Mm.eg.db (v3.13.0) was used for the genome‐wide annotation. Feature genes were revealed using the FindMarkers function in Seurat (using a Wilcoxon Rank Sum test, the log‐scale of the expression difference was set to 0.25). The criterion was set as an adjusted *p*‐value < .05.

### 5.10 Monocle trajectory analysis

HC trajectory analyses were performed with Monocle (R package v2.20.0; (http://cole‐trapnell‐lab.github.io/monocle‐release)), and pre‐processed Seurat objects were imported into Monocle using the “importCDS” function. Size factors and dispersions were pre‐calculated. Genes used to define a cell's progress were generated using the differentialGeneTest function. We then used Monocle's “orderCells” function to arrange cells along a pseudotime axis. Dot plots and heatmaps were used to visualize the highly variable genes’ expression trends along the pseudotime trajectory.

### 5.11 Identification of developmental trajectories

The trajectories were constructed using the Slingshot wrapper implemented in the dyno package (R packages v0.1.2(https://github.com/dynverse/dyno)). All parameters were left at default settings. The dynplot package (R packages v1.1.2(https://github.com/dynverse/dynplot)) was used to plot the trajectory within a scatterplot and to plot the relative gene expression level along the trajectory.

### 5.12 RNA velocity estimation

RNA velocity estimation was implemented using the scVelo python package (v0.2.4; https://github.com/theislab/scvelo) based on python version 3.9.7 (using conda environment, v4.10.3). We extracted spliced and unspliced reads using the standard velocyto pipeline (v0.17.17; https://github.com/velocyto‐team/velocyto.py), which exported a loompy file. The file was read into an AnnData object for downstream analysis using Scanpy (v1.8.1; https://github.com/theislab/scanpy). Here we referenced the recommended workflow of scvelo (http://velocyto.org). Our analysis procedures included four steps: (1) data pre‐processing, (2) implementing the dynamical model to determine the full transcriptional dynamics of splicing kinetics, (3) recovering the latent time based on transcriptional dynamics and (4) projecting the latent time onto the UMAP embedding that was exported from the Seurat analysis mentioned above.

### 5.13 Statistical analyses

SPSS 21.0 was used for all statistical analyses. Data are presented as the mean ± standard deviation (SD). We used student's *t*‐test to compare two groups or one‐way ANOVA followed by Dunnett's post hoc test to analyse more than two groups. Differences were considered statistically significant when *p* < .05.

## CONFLICT OF INTEREST

The authors declare no conflict of interest.

## Supporting information

Supporting Information

Supporting Information

Supporting Information

Supporting Information

Supporting Information

Supporting Information

Supporting Information

Supporting Information

Supporting Information

Supporting Information

Supporting Information

Supporting Information

Supporting Information
