# Supplementary figures and images for: The heterogeneity of mammalian utricular cells over the course of development
Source: Clin Transl Med. 2022 Sep 30;12(10):e1052. doi: 10.1002/ctm2.1052 (PMC9523683; doi:10.1002/ctm2.1052)

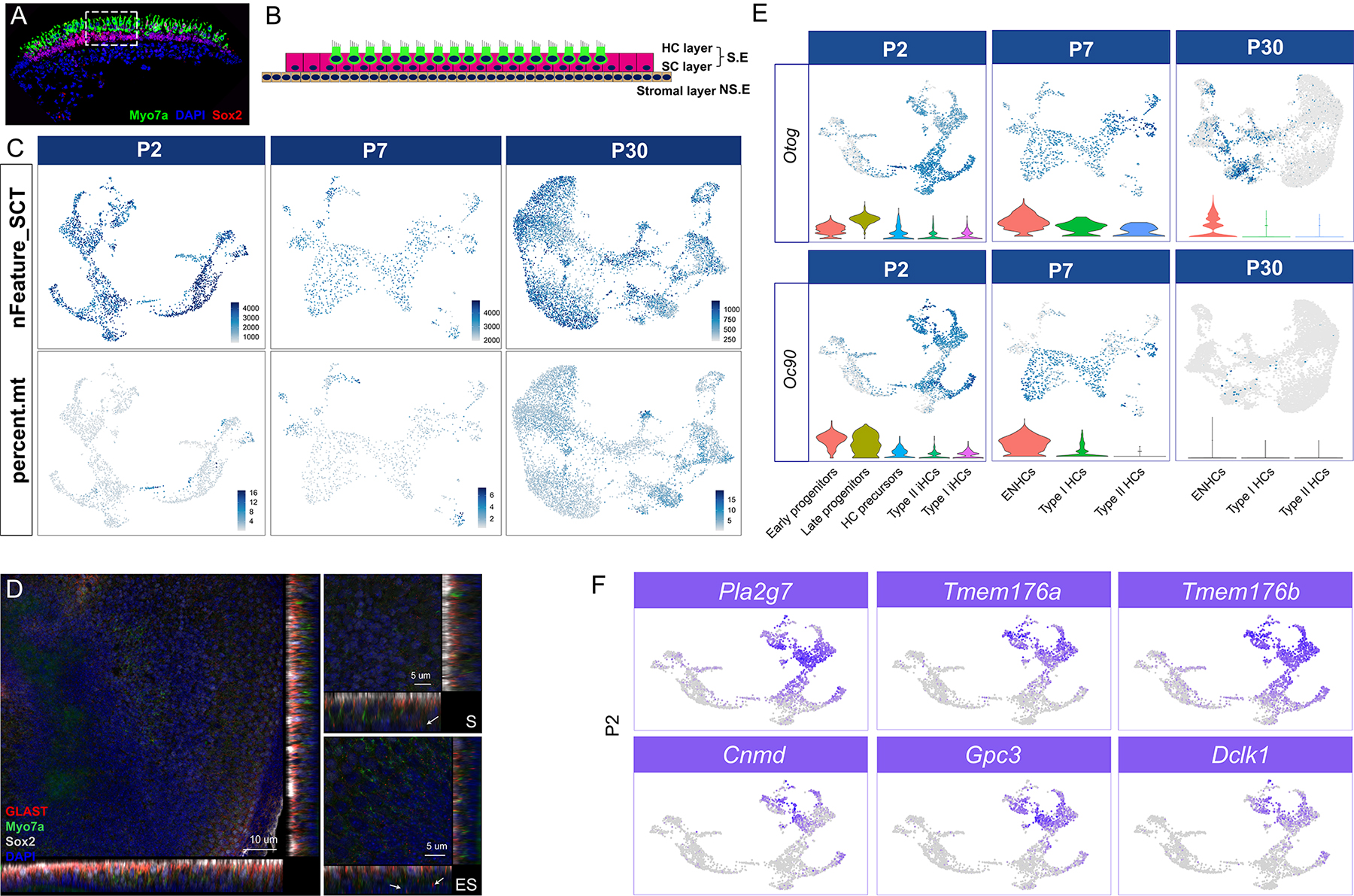

Supplement: Supplementary file 1 — Supporting Information [file CTM2-12-e1052-s008.jpg]

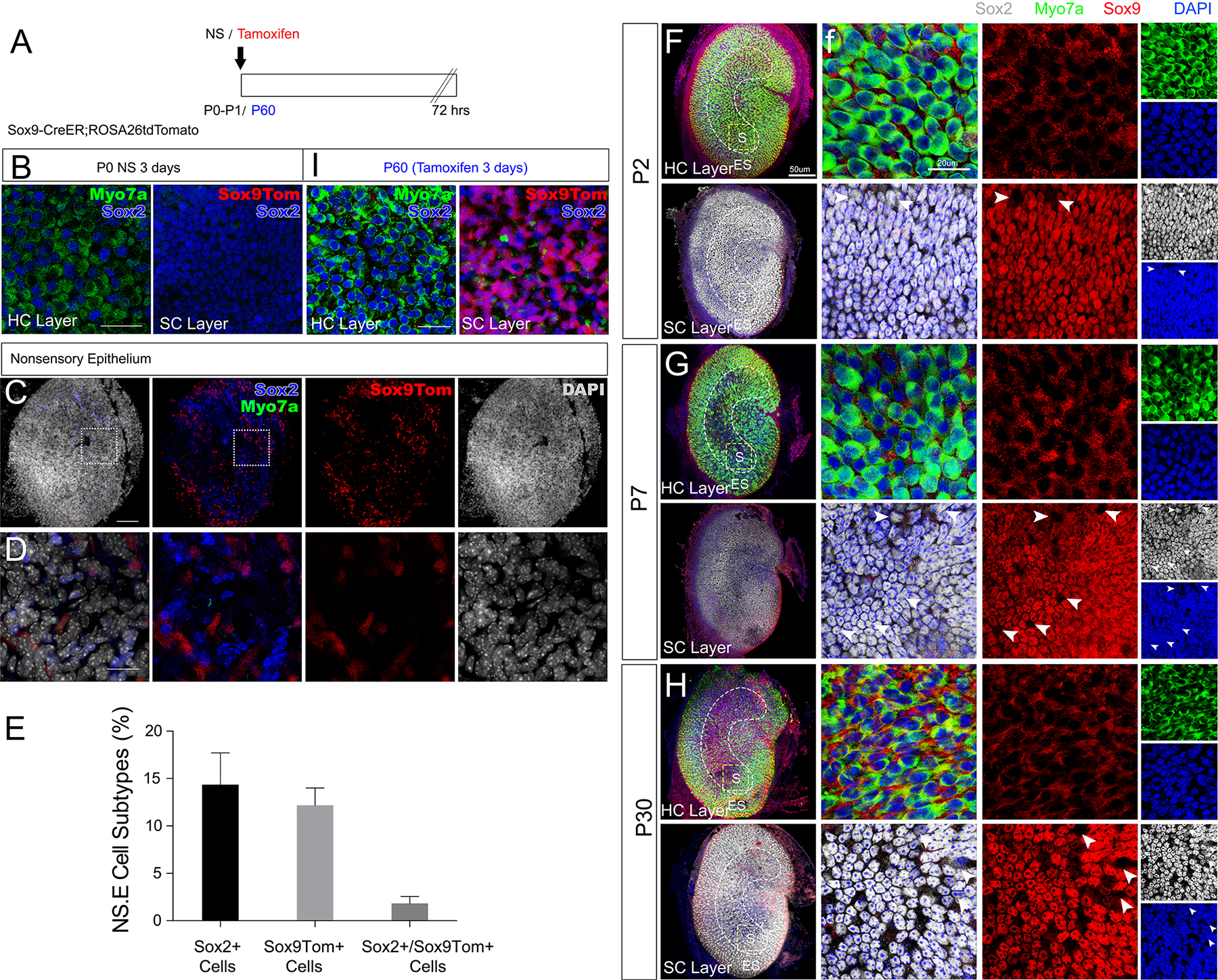

Supplement: Supplementary file 2 — Supporting Information [file CTM2-12-e1052-s007.jpg]

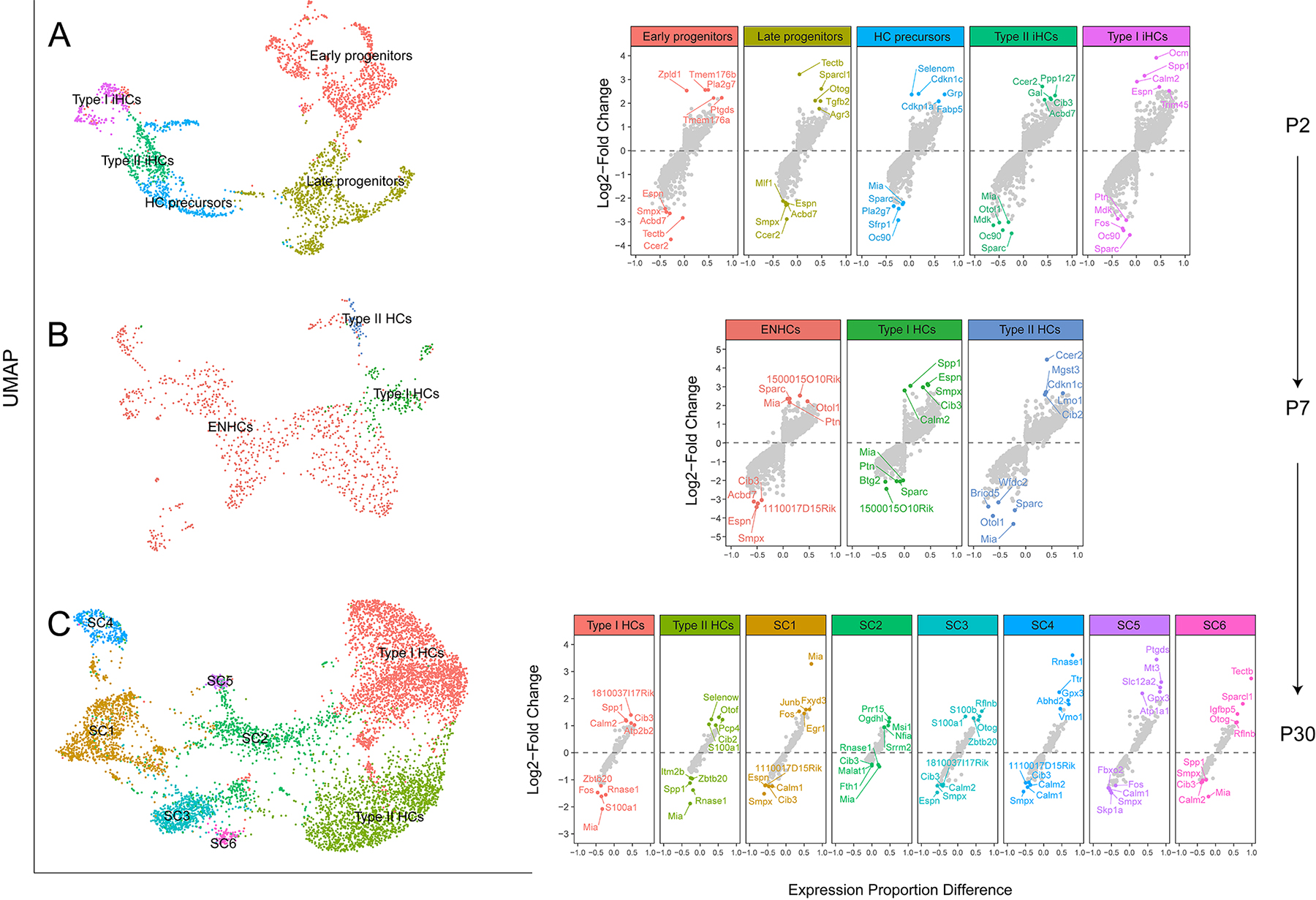

Supplement: Supplementary file 3 — Supporting Information [file CTM2-12-e1052-s009.jpg]

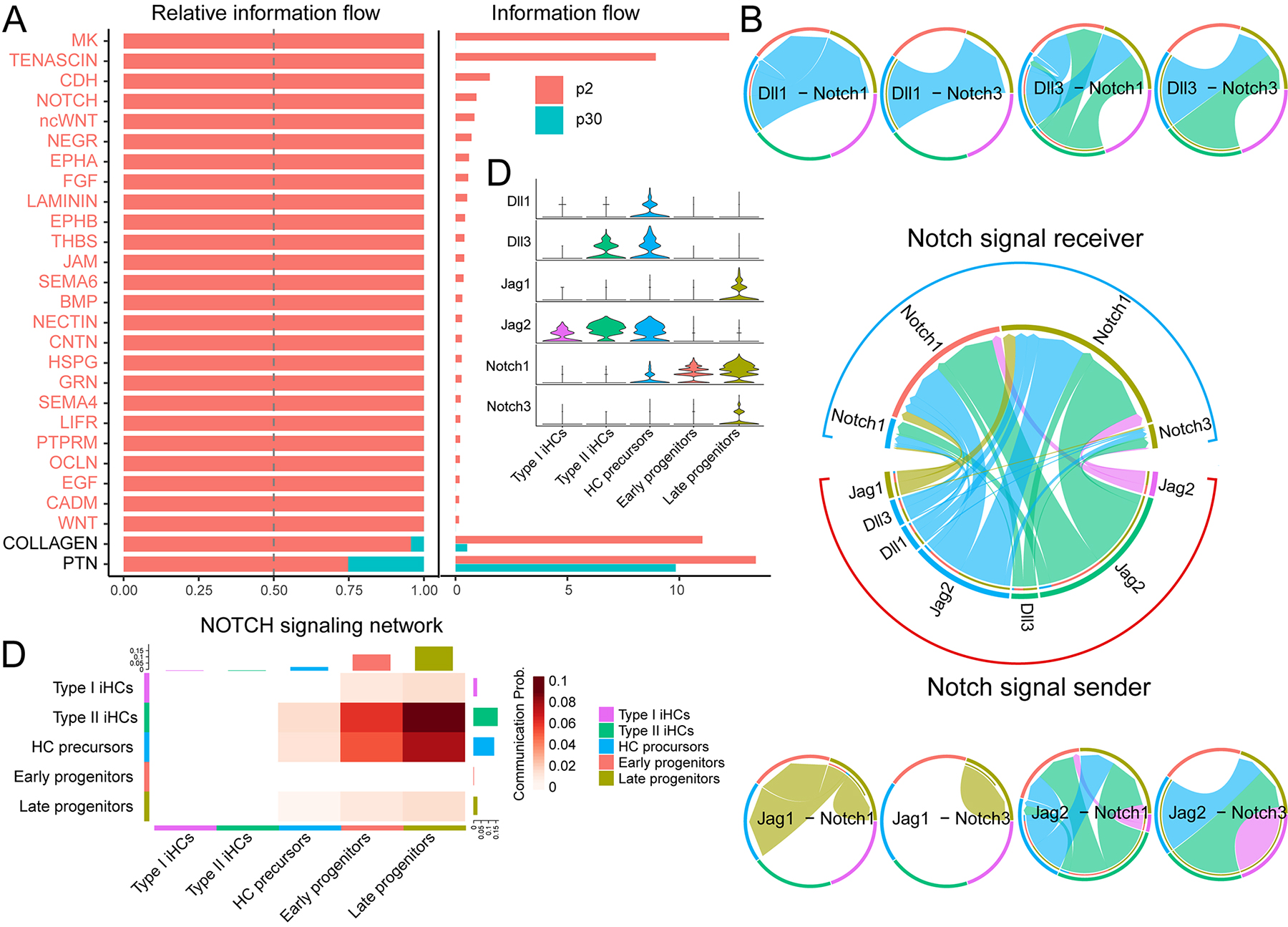

Supplement: Supplementary file 4 — Supporting Information [file CTM2-12-e1052-s006.jpg]

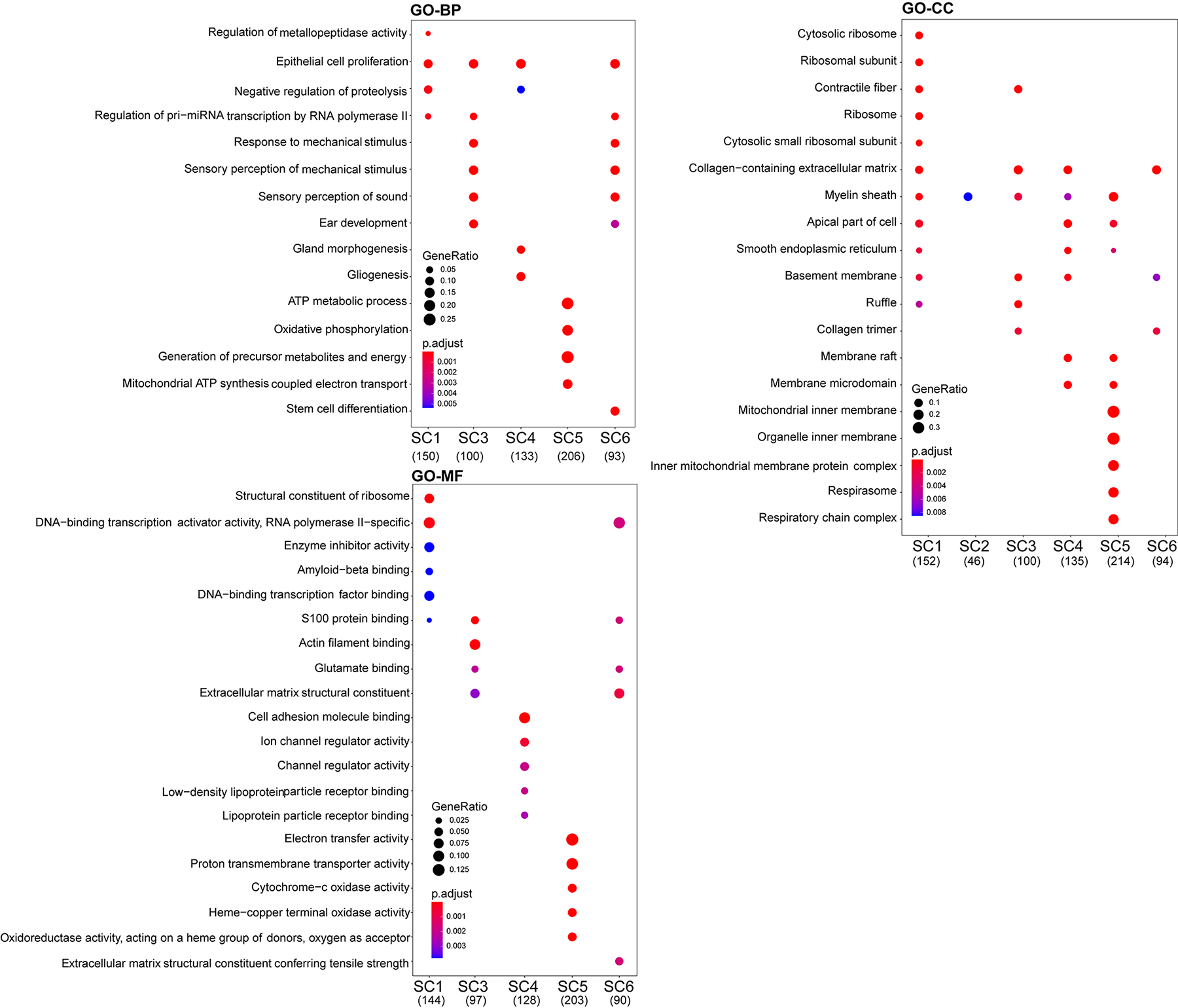

Supplement: Supplementary file 5 — Supporting Information [file CTM2-12-e1052-s002.jpg]

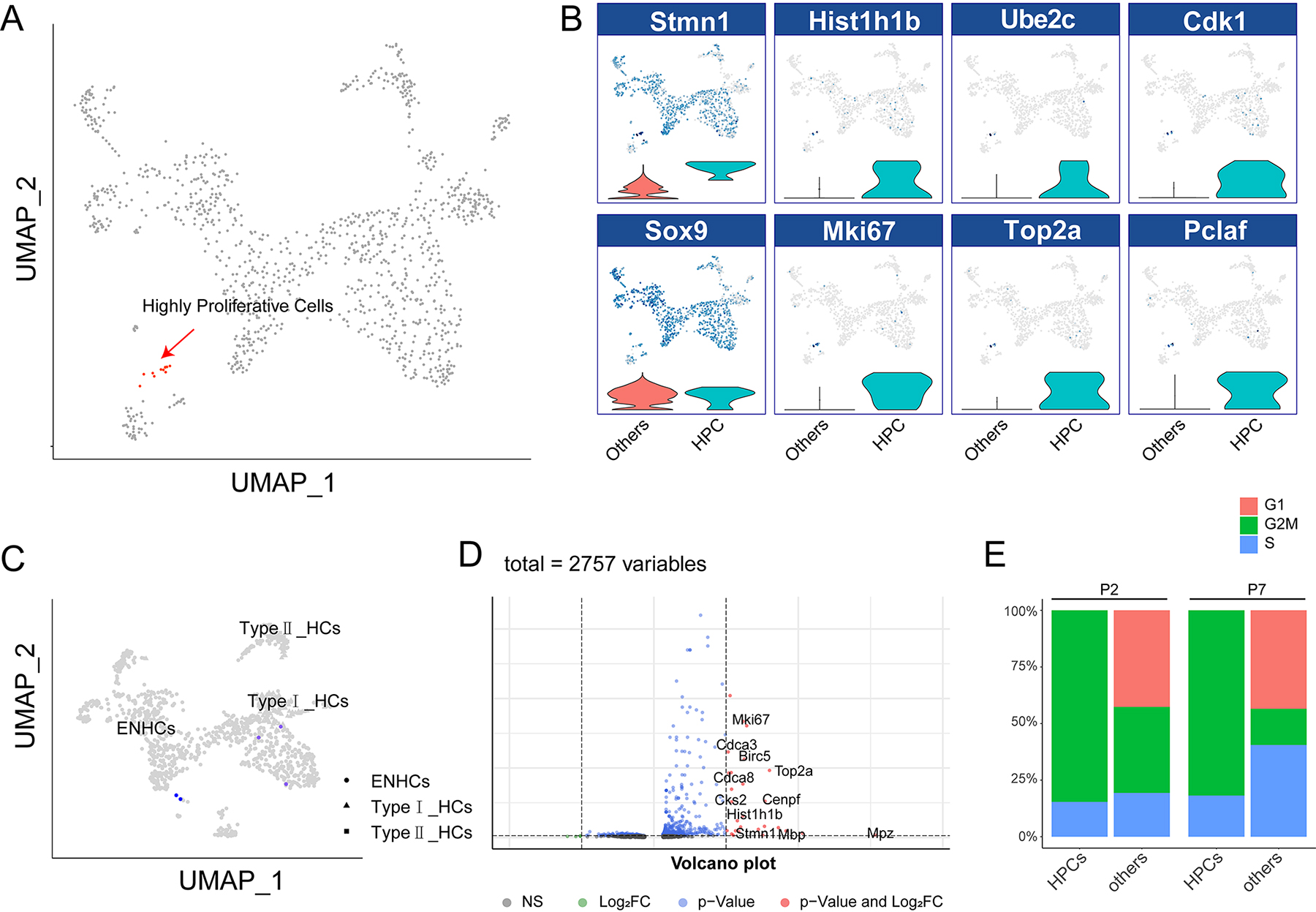

Supplement: Supplementary file 6 — Supporting Information [file CTM2-12-e1052-s001.jpg]

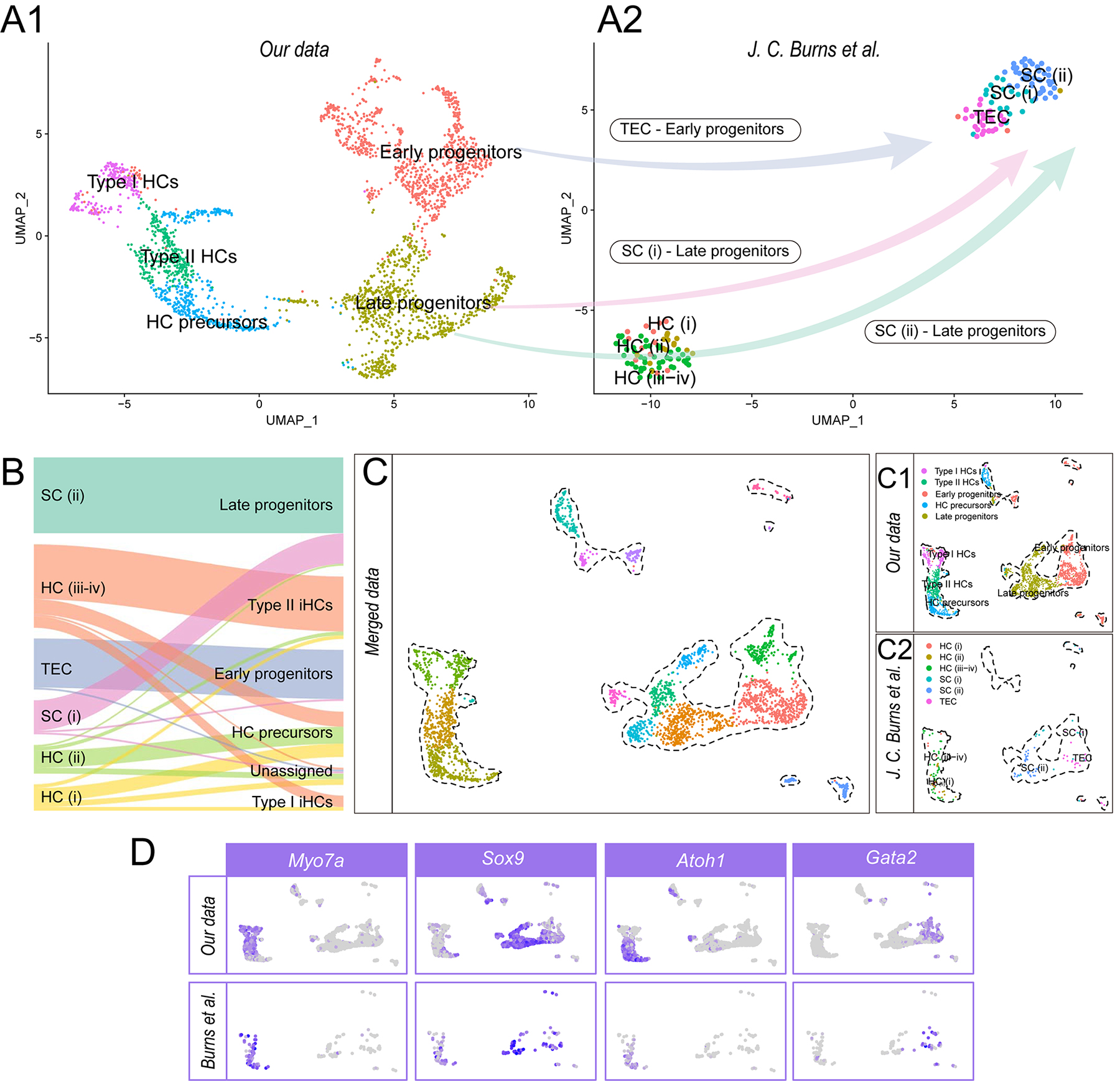

Supplement: Supplementary file 7 — Supporting Information [file CTM2-12-e1052-s004.jpg]

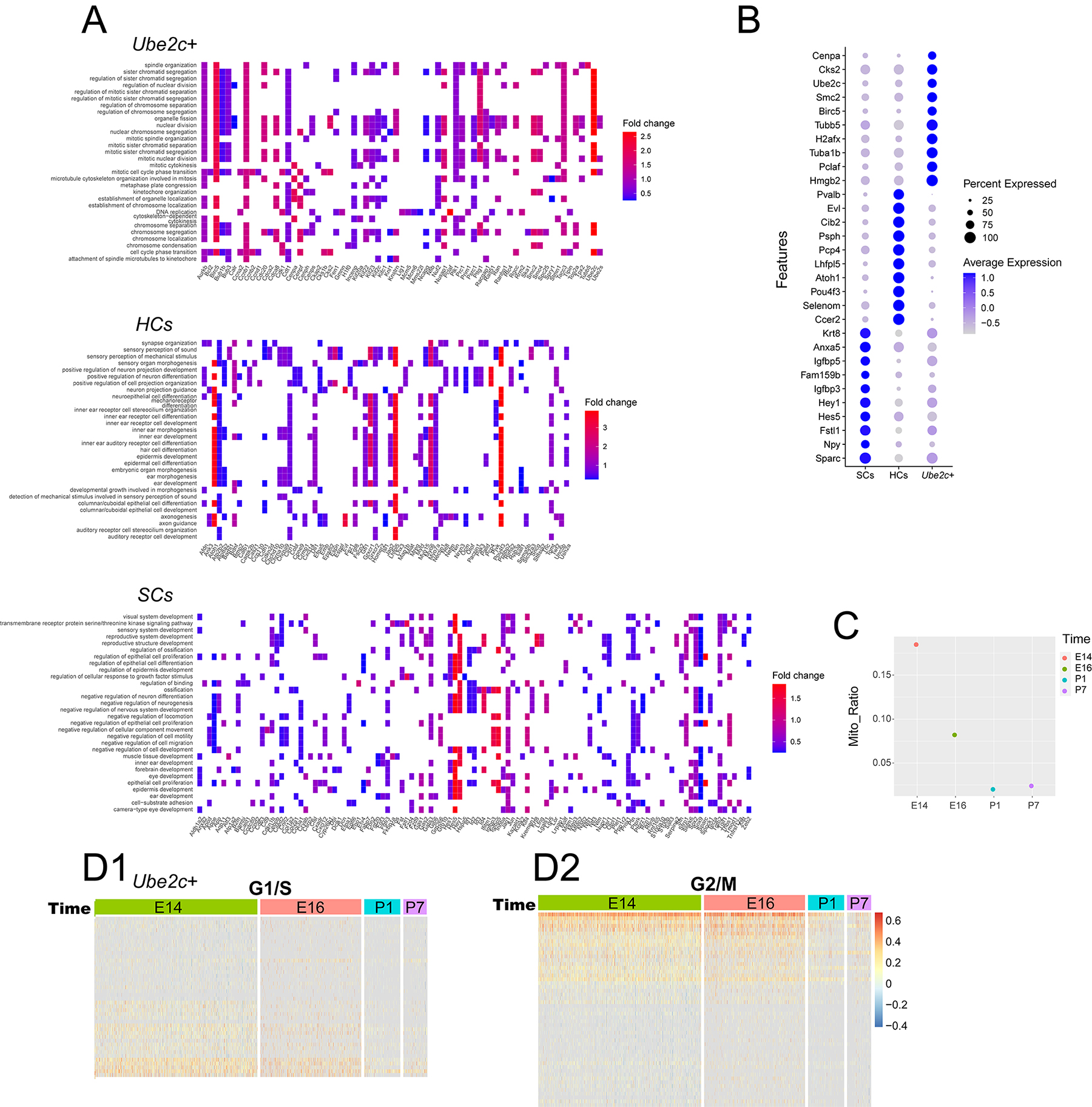

Supplement: Supplementary file 8 — Supporting Information [file CTM2-12-e1052-s012.jpg]
