# Supplementary material for: The heterogeneity of mammalian utricular cells over the course of development
Source: Clin Transl Med. 2022 Sep 30;12(10):e1052. doi: 10.1002/ctm2.1052 (PMC9523683; doi:10.1002/ctm2.1052)
Supplement: Supplementary file 9 — Supporting Information [file CTM2-12-e1052-s013.docx]

**Supplementary Figures**

**Figure S1. Data quality control and expression of Sox2, Myo7a, and GLAST. A-B)** Immunofluorescence image and diagram illustrating the sensory epithelium (S.E.) and non-sensory epithelium (NS.E.) of the mouse utricle. **C)** Quality control at time points P2, P7, and P30. UMAP plots showing the percentage of mitochondrial genes and the number of features in each cell. **D)** Slc1a3 (GLAST) expression in both the sensory epithelium and the stromal layer as indicated by the white arrows. **E)** Feature plots and violin plots showing the expression of Otog and Oc90 at three separate points. **F)** Genes expression in early progenitor cells in the P2 mouse utricle other than *Gata2*. Scale bars are 10 μm and 5 μm in D.

**Figure S2: Sox9 expression in sensory and non-sensory domains. A) A** schematic diagram illustrating the experimental procedure of neonatal (P0) or adult (P60) mice after normal saline (NS) or tamoxifen injection. **B)** NS was given at P0, and the utricles were harvested 72 h after administration. **C-E)** Expression of the *Sox9* and *Sox2* genes in the non-sensory epithelium. Sox2+ cells accounted for 14.33 ± 1.96% and Sox9tdTomato+ cells accounted for 12.16 ± 1.07% of the total cells in the epithelium that surrounds the sensory epithelium, while Sox2+/Sox9tdTomato+ cells accounted for only 1.81 ± 0.43%. F-H) Expression of *Sox9* in the hair cell layer and supporting cell layer in the P2, P7, and P30 mice utricle. White arrowheads show Sox2+/Sox9- cells. **I)** Tamoxifen was given_­­­_ at P60, and the utricles were harvested 72 h after administration. Scale bars are 50 μm in C and F and 20 μm in B, D, f, and I.

**Figure S3. Expression of cell type-specific markers across all clusters called in the UMAP plot. A)** P2**; B)** P7**; C) P**30.

**Figure S4. Cell-cell communication analysis of P2 utricle sensory epithelium cell subsets. A)** Comparison of the information flow between utricle sensory epithelial cell subclusters at different time points (P2 and P30). The bar plot on the left shows the relative cell-cell communication strength, while the bar plot on the right compares the absolute values of information flow. **B)** Chord diagrams visualizing the cell-cell communication network focusing on the Notch signaling pathway in the P2 utricle sensory epithelial cell subsets. The arrows point to potential signal recipient cells. The upper figure shows the underlying Notch-Dll signaling pathway, the lower figure shows the underlying Notch-Jag signaling pathway, and the central figure shows the Notch signaling pathway network with two arcs corresponding to signal senders and signal receivers. **C)** Violin plot of potential ligand and receptor gene expression related to the Notch signaling pathway. **D)** Hierarchy plot comparing the communication probability of the Notch signaling pathway between P2 utricle sensory epithelial cell clusters.

**Figure S5. Gene ontology (GO) analyses of SC clusters at P30.** GO subitems specifying the biological processes (GO-BP), molecular functions (GO-MF), and cellular components (GO-CC) of each SC cluster at P30 are shown.

**Figure S6. Potential mitotic generation of cells in P7 mouse utricle sensory epithelium. A)** UMAP clustering of cells from P7 mouse utricle sensory epithelium. **B)** Feature plots visualizing the marker genes of highly proliferative cells with feature plots and violin plots. **C)** Subtraction of cell cycle genes in the Ube2c+ subpopulation. ENHCs: epithelial non-HCs. **D)** Volcano plot showing the differentially expressed genes between highly proliferative cells and other cells in the P7 sensory epithelium (log2(fold-change) cut-off set to 2, *p*-value cut-off set to 0.001). **E)** Stacked bar chart comparing the cell cycle score between highly proliferative cells and other cells in the P2 and P7 sensory epithelium.

**Figure S7: Comparison of cell clusters between the present study and those from previous metadata. A1**) High-throughput single-cell RNA-seq results acquired by us. Uniform Manifold Approximation and Projection (UMAP) clustering of HCs, progenitors, and precursors from P2 mouse utricle sensory epithelium. Cells are colored by cell type. **A2**) Transcriptomes of 158 single cells from P1 mouse utricle sensory epithelium acquired by J. C. Burns et al. in 2015. Cells were captured using integrated fluidics circuit chips based on fluorescent protein tags. Uniform Manifold Approximation and Projection (UMAP) was used for dimension reduction. Cell types were defined following the cell metadata results reported by the authors (GSE71982). **B)** Projection of the single cell data sets acquired by J. C. Burns et al. onto our data set based on identified cell clusters. The connection between TEC and early progenitors, between SC (i) and late progenitors, and between SC (ii) and late progenitors were further labeled on fig A1&A2, C) Harmony integration results of the single cell transcriptomes of newborn utricle sensory epithelium acquired with two different methods. **C1-2**) The joint analysis of the two datasets shows the consistency of the single cell transcriptomes acquired by the two methods. **C1**) Categorization of our sequencing work from the integrated sample. Cells were labeled using our previous cluster definition. **C2**) Categorization of the sequencing samples acquired by J. C. Burns et al. Cells were labeled following cell metadata information. D) Featureplot showing marker gene expression patterns. The dotplots were split by different sample sources.

**Figure S8. Characteristics of the Ube2c+ cells in the cochlea (Data from Burns *et al,* 2015).** **A)** Heatmap showing the GO analysis of highly expressed marker genes from SCs, HCs, and Ube2c+ cells. Rows represent GO terms, and columns show the genes in each GO term. Color indicates the fold change of the marker genes. **B)** Dot plot showing the top 10 highly expressed marker genes of SCs, HCs, and Ube2c+ cells from E14, E16, and P1 mouse cochleae. **C)** The ratios of cycling cells at E14, E16, P1, and P7 by cell cycle analysis of mouse cochlear scRNA-seq (Burns et al. 2015). **D1-D2)** Heatmaps showing gene expression levels of G1/S **(D1)** and G2/M **(D2)** marker genes for each group of Ube2c+ cells in the mouse cochlea. Each column is one cell, and each row is a cell cycle gene. Red and blue represent increased and decreased gene expression, respectively, while grey represents no significant difference in gene expression.

**Supplemental Table 1: List for the antibodies used.**

| **Antibody** | **Source** | **Vendor** | **Dilution** | **Origin** |
| --- | --- | --- | --- | --- |
| Myosin VIIa | Rabbit Polyclonal | Proteus Biosciences | 1:800 | Ramona, CA, US |
| Myosin VIIa | Mouse Polyclonal | Santa Cruz | 1:500 | California, US |
| Sox2 | Goat polyclonal | Santa Cruz | 1:300 | California, US |
| Calbindin | Rabbit Polyclonal | EMD Millipore | 1:500 | Massachusetts, US |
| EdU | Fluorescent dye | Life Technologies | / | Carlsbad, CA, US |
| Stmn1 | Rabbit Polyclonal | Abcam | 1:500 | Cambridge, UK |
| UBCH10 | Rabbit Polyclonal | Abcam | 1:200 | Cambridge, UK |
| GLAST | Rabbit Polyclonal | Abcam | 1:500 | Cambridge, UK |
| Tecb | Rabbit Polyclonal | Life Technologies | 1:500 | Carlsbad, CA, US |
| Mki67 | Mouse IgG1 | Cell Signaling Technology | 1:500 | Massachusetts, US |
| DAPI | Fluorescent dye | Sigma-Aldrich | 1:800 | St. Louis, MO |
| Parvalbumin | Mouse Polyclonal | Sigma-Aldrich | 1:400 | St. Louis, MO |
| Sox9 | Polyclonal Goat IgG | R&D Systems | 1:300 | CA, US |
| Gata2 | Rabbit Polyclonal | Invitrogen | 1:200 | MN,USA |
|  |  |  |  |  |

**Legends for supplementary videos.**

**Video 1**. During the neonatal period, large numbers of proliferating ENHCs (co-labeled with red, white, and blue) were detected in both the striolar and extrastriolar regions (arrowheads).

**Video 2.** The proliferating ENHCs (co-labeled with red, white, and blue) captured at P2–P7 (arrowheads).

**Video 3.** The proliferating ENHCs (co-labeled red, white, and blue) captured at P7–P14 (arrowheads).

**Video 4.** Turnover of HCs derived from the differentiation of ENHCs/SCs (co-labeled green, red, and blue) is mainly found in the extrastriolar region 3 weeks after birth.
